# Global research trends and emerging opportunities for integrin adhesion complexes in cardiac repair: a scientometric analysis

**DOI:** 10.3389/fcvm.2024.1308763

**Published:** 2024-04-18

**Authors:** Jiayu Lv, Zhenyue Fu, Haoran Zheng, Qingqiao Song

**Affiliations:** ^1^ Department of General Internal Medicine, Guang’anmen Hospital, China Academy of Chinese Medical Sciences, Beijing, China; ^2^ China Academy of Chinese Medical Sciences, Beijing, China; ^3^ College of Traditional Chinese Medicine, Beijing University of Traditional Chinese Medicine, Beijing, China

**Keywords:** integrins, cardiac repair, cardiac regenerative medicine, bibliometrics, myocardial infarction

## Abstract

**Objective:**

Cardiac regenerative medicine has gained significant attention in recent years, and integrins are known to play a critical role in mediating cardiac development and repair, especially after an injury from the myocardial infarction (MI). Given the extensive research history and interdisciplinary nature of this field, a quantitative retrospective analysis and visualization of related topics is necessary.

**Materials and methods:**

We performed a scientometric analysis of published papers on cardiac integrin adhesion complexes (IACs), including analysis of annual publications, disciplinary evolution, keyword co-occurrence, and literature co-citation.

**Results:**

A total of 2,664 publications were finally included in the past 20 years. The United States is the largest contributor to the study and is leading this area of research globally. The journal *Circulation Research* attracts the largest number of high-quality publications. The study of IACs in cardiac repair/regenerative therapies involves multiple disciplines, particularly in materials science and developmental biology. Keywords of research frontiers were represented by Tenasin-C (2019–2023) and inflammation (2020–2023).

**Conclusion:**

Integrins are topics with ongoing enthusiasm in biological development and tissue regeneration. The rapidly emerging role of matricellular proteins and non-protein components of the extracellular matrix (ECM) in regulating matrix structure and function may be a further breakthrough point in the future; the emerging role of IACs and their downstream molecular signaling in cardiac repair are also of great interest, such as induction of cardiac proliferation, differentiation, maturation, and metabolism, fibroblast activation, and inflammatory modulation.

## 1 Introduction

The study of integrins dates back to the early 1970s (1). As heterodimeric receptors on the surface of cell membranes, integrins and integrin-mediated adhesion perform a “scaffolding function” for physical anchoring between cells and the extracellular matrix (ECM). More importantly, they are recognized to sense the biochemical and biophysical properties of the microenvironment, serve as bidirectional hubs transmitting and translating signals, and respond by activating signaling networks that regulate cell structure, dynamics, behavior, and fate, thereby coordinating a wide range of life activities in organisms, such as organogenesis, tissue repair, matrix remodeling, and immune response, and regulating cell phenotype, proliferation, growth, differentiation, migration, and survival (2, 3). To date, integrins are well known to be involved in various diseases, such as platelet disorders, atherosclerosis, cancer, osteoporosis, fibrosis, renal diabetic neuropathy, and macular degeneration. After the 1970s, with the progress of monoclonal antibody technology and the paradigm shift towards target-led drug discovery, a number of effective marketed therapies targeting integrins were developed accordingly (4, 5).

As for cardiovascular health, integrins and integrin adhesion complexes (IACs) play a crucial role in cardiac development, repair, and remodeling. These include embryonic morphogenesis, myocardial proliferation, hypertrophic growth, ion channels, contractility, fibrosis, ischaemic stress, and heart failure (HF). During the postnatal period, mammalian cardiomyocytes undergo a maturational transition that manifests as mitotic arrest, multinuclear/polyploid, and hypertrophic growth (6). Cardiomyocyte loss during myocardial infarction (MI) is usually followed by fibrous scar formation and compensatory changes in distal heart tissue, ultimately leading to HF and premature death. Based on current evidence, the adult heart retains a certain ability to generate new cardiomyocytes, and the genesis of cardiomyocytes occurs by division of pre-existing cardiomyocytes during normal aging as well as after myocardial injury (7, 8); however, this rate of cardiomyocyte renewal is very low, showing an age-related decline, and an insufficiency in repairing myocardium after myocardial injury, although it is enhanced upon MI (9, 10). As a result, research for cardiac regeneration has been widely carried out as an ideal therapeutic strategy to replace lost cardiomyocytes, including cell transplantation or therapies for promoting endogenous regenerative processes (10). There is growing evidence that regeneration involves the reconstitution of multiple cell types and structures following cardiac injury rather than the repair or regeneration of myocardial tissue by reconstituting cardiomyocytes alone and that coordination of connections and communication between cardiomyocytes and with non-cardiomyocytes and the extracellular matrix is essential (11). Integrins and constituents of integrin adhesome (e.g., integrin-linked kinase, focal adhesion kinase) act as critical contributors to cardiac reconstitution of functional and structural tissue, such as promoting the proliferation, differentiation, and maturation of cardiomyocytes and angiogenesis, and improving the microenvironment associated with inflammation and oxidative stress and fibrosis as well as modulating biochemical and mechanical signaling of extracellular matrix origin, demonstrating potential as effective targets and theoretical support for cardiac regeneration. Multiple cardiac regenerative therapies based on these, like gene therapy, cell transplantation, injectable delivery matrices, and cardiac tissue engineering, have received increased attention in the last 20 years, particularly in cardiac repair.

Yet, despite great enthusiasm and positive preclinical results, clinical translation of cardiovascular regenerative/repair therapies has not yet been successful, and currently accepted priority options focus on uncovering potential pathways and control mechanisms of cardiac regeneration (11). Given the significant impact of integrins on cardiac development and repair and the abundance of research conducted in the past two decades (5), there is a pressing need to compile essential evidence in this area. Furthermore, integrins and their associated models are conceptually diverse and involve various subject knowledge, accompanied by a continuous update of complex biological mechanisms, such as bidirectional mechanotransduction, spatiotemporal-specific expression, inter-crosstalk with ECM components, composition and assembly of IACs, and activation of specific signaling pathways. These have significantly complicated the current status of the corresponding structural domains, posing additional difficulties for the development of their research and clinical translation in specific problems of cardiovascular regeneration. In this analysis, we aim to provide a clearer picture based on scientometrics regarding the evolution of the knowledge structure and hotspots of integrins in cardiac repair. This information can provide a more comprehensive and in-depth insight into the current state and frontiers of the field, uncover opportunities and challenges, and facilitate the clinical translation of strategic decisions on integrins and their related compounds in cardiac regenerative medicine research.

## 2 Methods

### 2.1 Systemic search strategy

Web of Science (WoS) was founded in 1997 and is currently the largest comprehensive academic information resource database globally, covering most disciplines. To ensure the quality and accessibility of the data, we focus on the Web of Science Core Collection (WoSCC) database. WoS's topic search is equivalent to a keyword search of some fields, including title, abstracts, author keywords, and keywords plus. To capture the topics more accurately, we used the topics “cardiac remodeling,” “cardiac hypertrophy,” “cardiac fibrosis,” “myocardial infarction,” “Cardiomyocyte apoptosis,” “Heart failure,” “Cardiac repair” and “Integrin” as searches in SCIEXPANDED, SSCI, A&HCI, ESCI, CCR-EXPANDED, ic and a search of the literature for the period 1 January 2003–21 March 2023. After searching as described above, a total of 2,768 records were obtained.

### 2.2 Eligibility criteria

**Figure 1:**
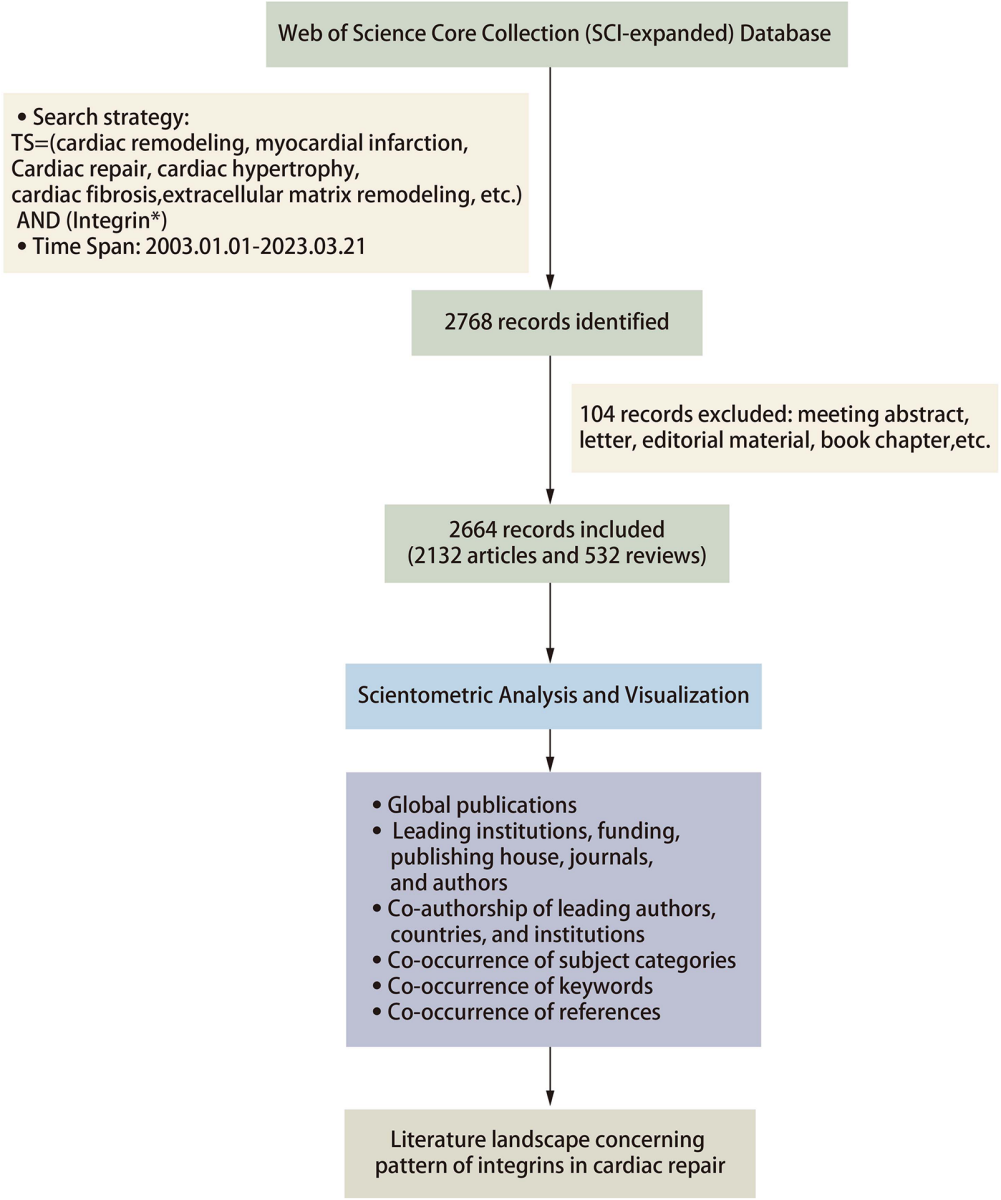
Flowchart of the analysis methods.

The inclusion criteria for the database were limited to studies that met the following conditions: (1) article and review article indexed in the database, (2) published and had sufficient data for analysis, (3) written in English, and (4) relevant to the research content. Any studies that did not meet these criteria were excluded from the analysis. In total, we filtered the literature to be searched and finally included 2,664 records for quantitative analysis. Figure 1 depicts the detailed process of publications inclusion and exclusion.

### 2.3 Scientometric and visualized analysis

For this scientometric analysis, Citespace (version 5.8.R5W) and Vosviewer software were selected as the primary tools for visual analysis of the literature in the present review. The data were imported into Citespace software and first checked for duplicates. Running the deduplication program showed no duplicates in the literature included in this study. Regarding the parameter function selection, the selection time is from 2003 to 2023 with a 1-year time slice. In Vosviewer, we analyzed the keywords and selected the minimum number of documents of a node based on the need to visualize the data and set the others to default values. In Citespace, we analyzed the references and used the pathfinding pruning method and minimum spanning tree algorithm to simplify the network and highlight basic features. We set the others as default values in Citespace. Betweenness centrality (BC) is calculated to evaluate the strength of a node connection with other nodes. Burst indicates a sharp change in a short period; larger nodes mean a higher frequency of occurrence; a thicker line between nodes implies a stronger correlation of node co-occurrence or co-citation. GraphPad Prism 8, Scimago Graphica, and Origin 2021 are also involved in literature visualization for a more precise presentation of the data (Figure 1).

## 3 Results

### 3.1 Trend of global publications

**Figure 2:**
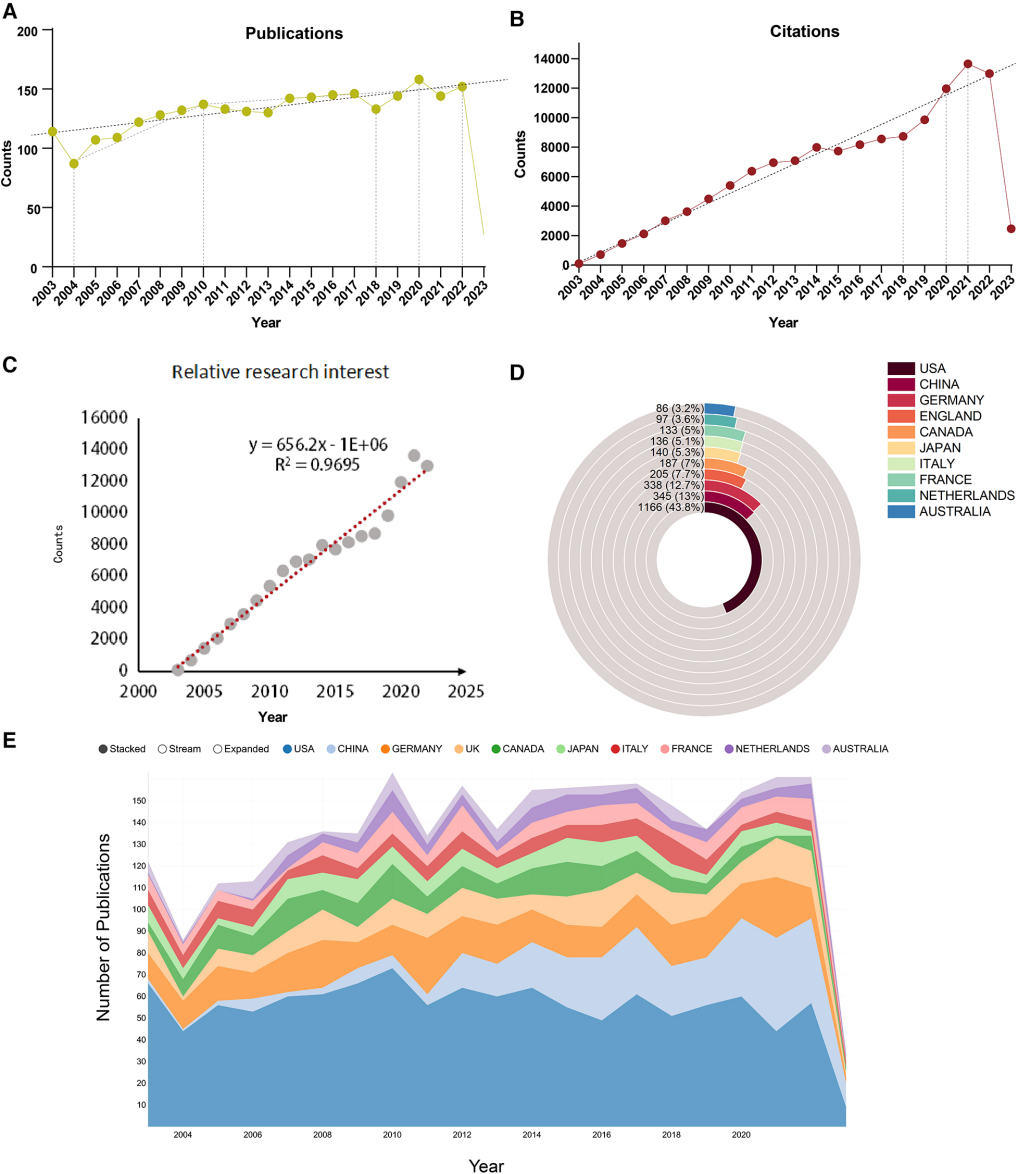
Global trends and countries/regions contributing to the field of IACs in cardiac repair (2003–2023). (**A**) The annual number of publications; (**B**) The annual number of citations; (**C**) Model fitting curves of global trends in citations; (**D,E**) The sum of publications in the top 10 countries/regions.

From 2003 to 2023, a total of 2,664 publications on the subject of cardiac integrins were published in the WoSCC, including 2,132 articles (80.03%) and 532 reviews (19.97%). Citation reports from the WoS show that these publications have been cited a total of 133,380 times, with an average of 50.09 citations per article, and increasing each year. As shown in Figure 2A, the annual publication statistics show an overall increasing trend in the number of publications in the field yearly, although with fluctuations. The overall trend of annual publication volume can be divided into two phases: a six-year period of steady growth from 87 publications in 2004 to 137 publications in 2010 and a 12-year period of slow increase to 152 publications from 2010 to 2022. In particular, as shown in Figures 2A,B, while undergoing a slight decline in some years, such as 2013 and 2018, there was a linear increase in annual publications over the time span from 2018 to 2020, with the corresponding number of citations peaking at 13,651 times from 2018 to 2021, heralding potentially groundbreaking research. The relative research interest was defined as the number of citations in one certain field by all field publications per year. As shown in Figure 2C, a linear fitting model was constructed to predict the future global trend, and the fitting curve was *Y* = 656.2 × *X*−1E + 06 (*R*^2^ = 0.9695), indicating that global interests in this area in the coming years might increase at a stationary rate. More specifically, as shown in Figures 2C,D, the USA contributed mostly to this research topic and published the most papers (1,166, 43.8%) in the past 20 years, followed by China (345), Germany (338), England (205), and Japan (140). The yearly increase in published papers and citations indicates that cardiac integrins have been a subject of growing interest and attract wider attention from scholars.

### 3.2 Global publications analysis of leading institutions, funding, publishing houses, journals, and authors

**Figure 3:**
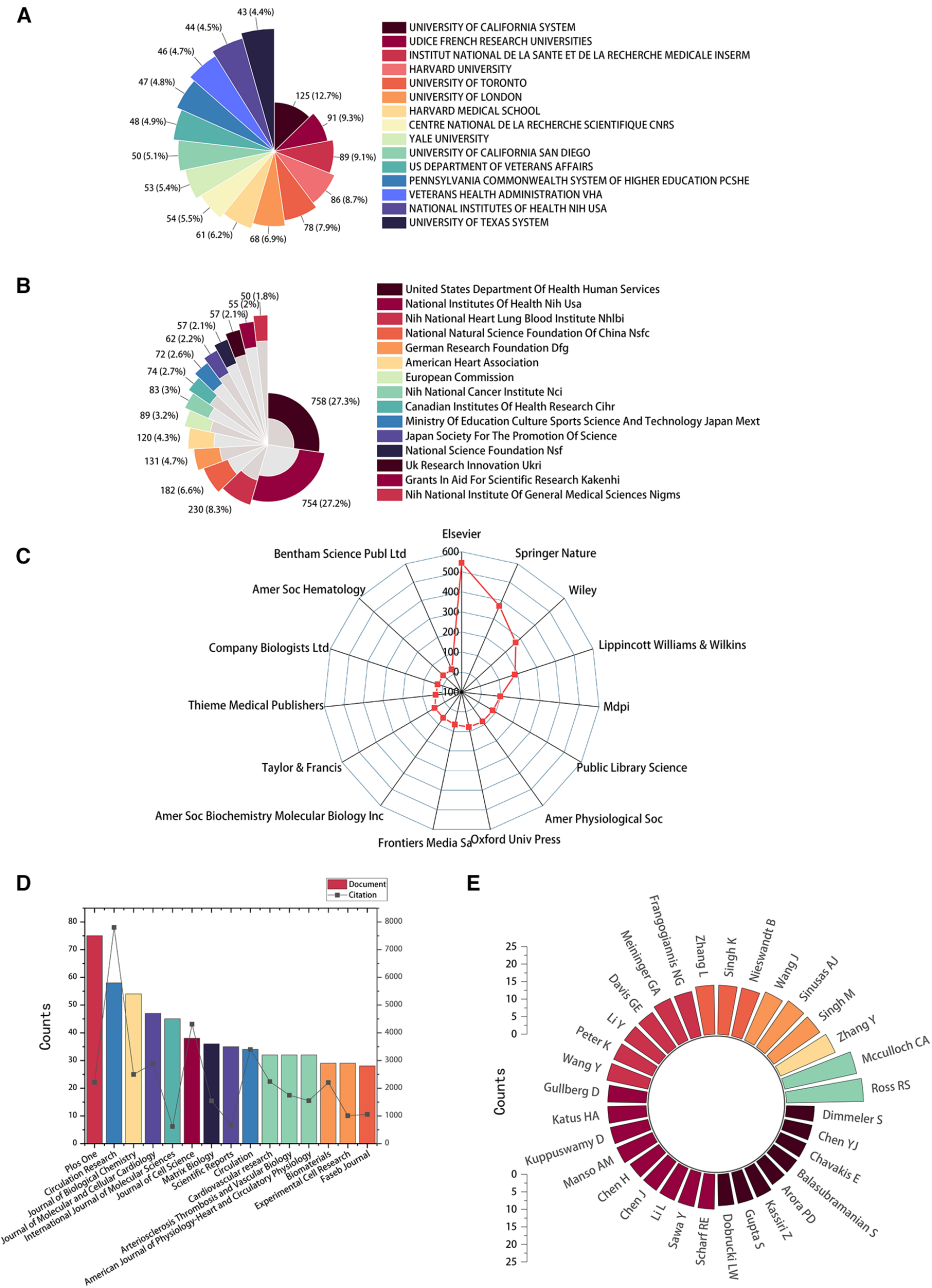
High-contribution institutions, funds, publishing houses, journals, and authors of global publications about IACs in cardiac repair (2003–2023). (**A**) The top 15 institutions with the most publications. (**B**) The top 15 funding sources with the most publications. (**C**) The top 15 publishing houses with the most publications. (**D**) The top 15 journals with the most publications. (**E**) The top 32 authors with the most publications.

The top 15 contributive institutions are listed in Figure 3A. University of California System from the USA published the most (125 publications), and Udice French Research Universities from France ranked second (91 publications). In contrast, Institut National De La Sante Et De La Recherche Medicale Inserm from France ranked third (89 publications). The top 15 funding sources are shown in Figure 3B. In total, 758 publications were funded by the United States Department of Health Human Services (Tied for first), 754 publications were funded by the National Institutes of Health (NIH, USA) (ranked second), and 203 publications were funded by National Heart Lung and Blood Institute (NHLBI) (ranked third). The top 15 publishing houses are shown in Figure 3C. Overall, Elsevier from the Netherlands ranked first with 544 publications, Springer Nature from Germany ranked second with 370 publications, and Wiley from the USA ranked third with 370 publications. As shown in Figure 3D and Table 1, *Plos One* [impact factor (IF) = 3.752, 2021] published the most with 75 publications. There were 58 publications in *Circulation Research* (IF = 23.213, 2021), with the most citations and the average number of citations per document. *Journal of Biological Chemistry* (IF = 3.862, 2021) published 54 documents, ranking third. Moreover, *Circulation* has the highest IF and H-index with 36 publications. We also produced two additional graphs to present the IF and number of publications of journals over time (2003–2023) (see Supplementary material Figures S1,S2). As shown in Figure 3E, Ross RS contributed the most research with 22 publications, followed by Mcculloch CA with 21 publications, and Zhang Y with 17 publications.

**Table 1:** The top 20 journals in the feld of cardiac IACs (2003–2023) by TD.

NO	Journal	TD	TC	ACD	IF (2021)	H-index	Publishing house
1	Plos One	75	2,214	29.52	3.752	332	Public Library of Science
2	Circulation Research	58	7,804	134.55	23.213	336	Lippincott Williams and Wilkins Ltd.
3	Journal of Biological Chemistry	54	2,498	46.26	3.862	101	American Society for Biochemistry and Molecular Biology Inc.
4	Journal of Molecular and Cellular Cardiology	47	2,888	61.45	5.763	159	Elsevier
5	International Journal of Molecular Sciences	45	615	13.67	6.208	162	Multidisciplinary Digital Publishing Institute (MDPI)
6	Journal of Cell Science	38	4,311	113.45	5.235	278	Company of Biologists Ltd
7	Matrix Biology	36	1,544	42.89	10.447	117	Elsevier
8	Scientific Reports	35	671	19.17	4.996	213	Nature Publishing Group
9	Circulation	34	3,399	99.97	39.918	607	Lippincott Williams and Wilkins Ltd.
10	Cardiovascular Research	32	2,239	69.97	13.081	219	Oxford University Press
11	Arteriosclerosis Thrombosis and Vascular Biology	32	1,746	54.56	10.514	207	Lippincott Williams and Wilkins Ltd.
12	American Journal of Physiology-Heart and Circulatory Physiology	32	1,550	48.44	5.125	197	American Physiological Society
13	Biomaterials	29	2,203	75.97	15.304	381	Elsevier
14	Experimental Cell Research	29	1,008	34.76	4.145	188	Elsevier
15	Faseb Journal	28	1,056	37.71	5.834	277	FASEB
16	Blood	23	2,170	94.35	25.476	465	American Society of Hematology
17	Thrombosis and Haemostasis	23	901	39.17	6.681	188	Georg Thieme Verlag
18	Frontiers in Cell and Developmental Biology	22	311	14.14	6.081	53	Frontiers Media S.A.
19	American Journal of Physiology-Cell Physiology	19	720	37.89	5.282	181	American Physiological Society
20	Stem Cells	18	913	50.72	5.845	299	Wiley-Blackwell

TD, the total number of documents; IF, impact factor; TC, the total number of citations; ACD, the average number of citations per document.

### 3.3 Co-authorship analysis of leading authors, countries, and institutions

**Figure 4:**
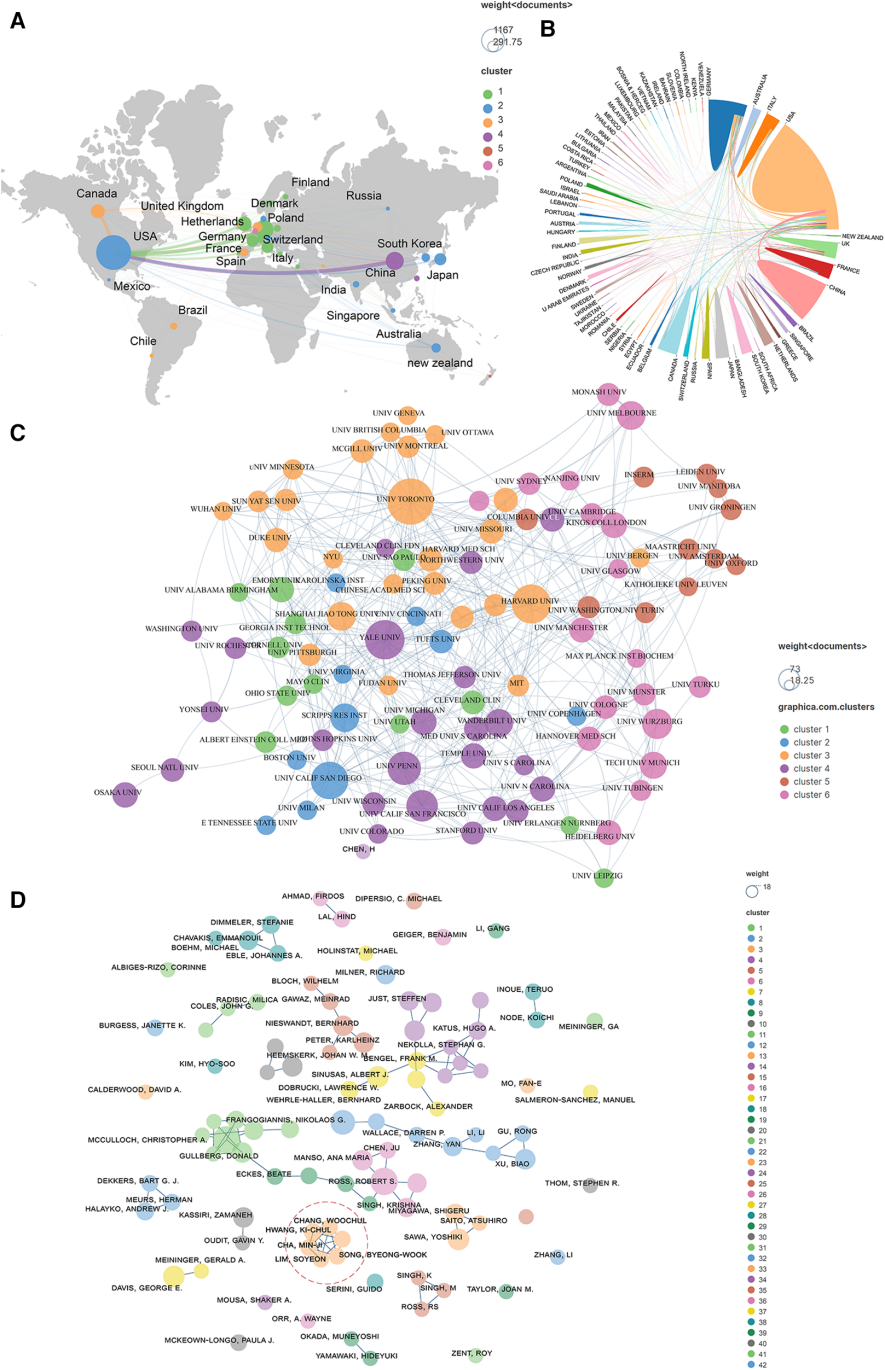
Co-authorship analysis of leading authors, countries, and institutions field of cardiac IACs (2003–2023). (**A,B**) Co-authorship analysis of the leading countries. (**C**) Co-authorship analysis of the 101 institutions. (**D**) Co-authorship analysis of the 101 leading authors. The line between different points represents that the authors/institutions/countries had established a cooperative relationship. The thicker the line, the stronger the link between the authors/institutions/countries. Red dashed area: top 5 authors in terms of collaborative intensity.

Publications (the minimum number of documents of each country is defined as more than 20) originating from 27 countries were analyzed via Vosviewer and visualized by Scimago Graphica (Figures 4A,B). Total link strength (TLS) represents the intensity of cooperation with other countries, institutions or authors. The top 5 countries with large TLS are as follows: USA (TLS equals 499), Germany (TLS equals 271), England (TLS equals 229), Italy (TLS equals 129), and Canada (TLS equals 128). One hundred organizations (the minimum number of documents used by an organization was defined as more than 12) were analyzed and visualized in Figure 4C. The top 5 institutions with the largest TLS are shown as follows: Harvard Univ (TLS equals 67), Univ Toronto (TLS equals 64), Univ Penn (TLS equals 42), Yale Univ (TLS equals 39), and Brigham and Womens Hosp (TLS equals 37). A total of 101 authors (the minimum number of documents of each country was defined as more than 5) were analyzed and visualized (Figure 4D). The top 5 authors in terms of collaborative intensity are shown below: Hwang, Ki-Chul (TLS equals 30), Cha, Min-Ji (TLS equals 27), Chang, Woochul (TLS equals 27), Lim, Soyeon (TLS equals 27), and Song, Byeong-Woo (TLS equals 27).

### 3.4 Co-occurrence analysis of subject categories

**Figure 5:**
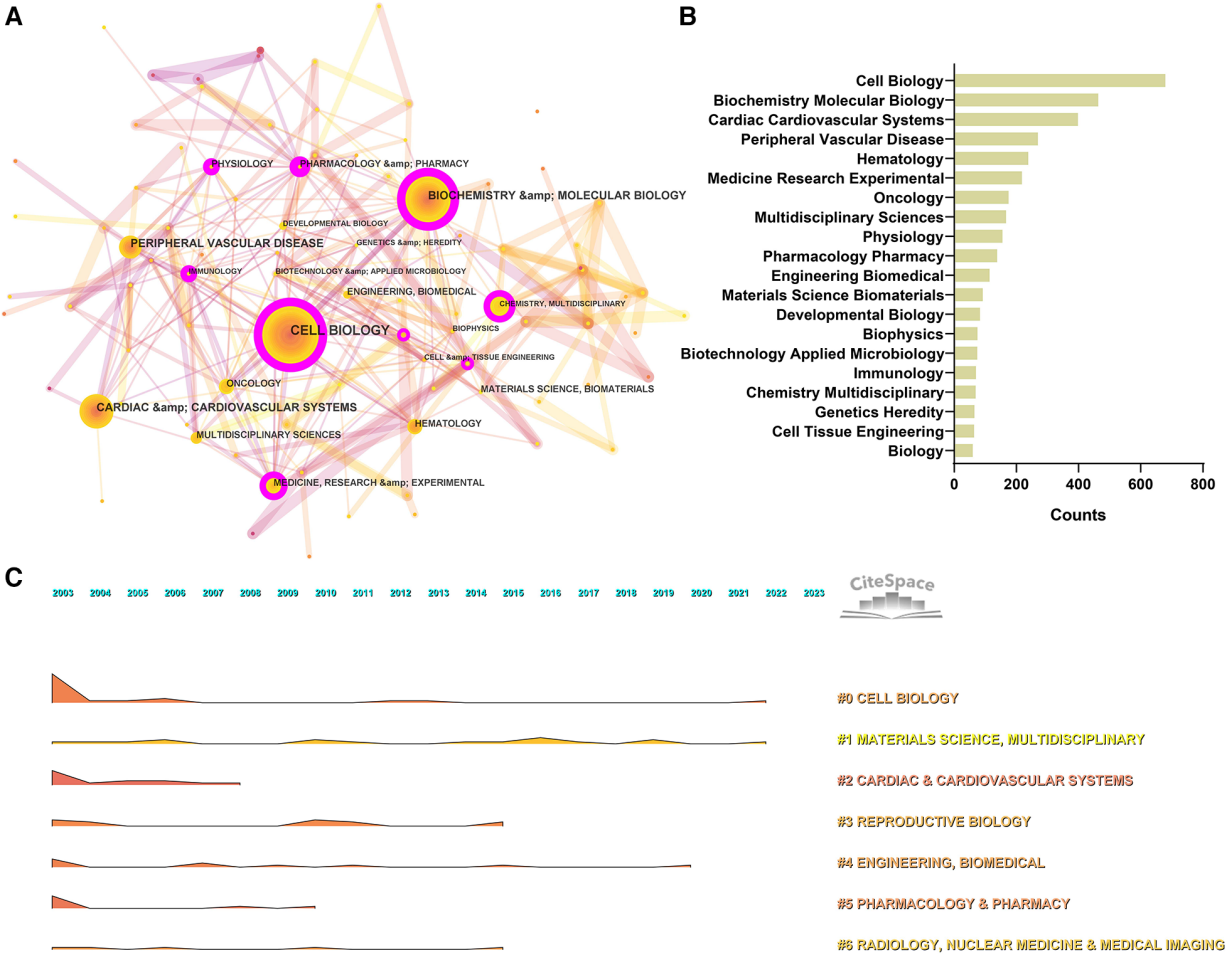
Co-occurrence analysis of subject categories in the field of cardiac IACs (2003–2023). (**A**) Disciplinary network. The node size represents the number of occurrences of the discipline. The larger the node, the more occurrences; the purple circle outside the node, BC > 0.1. (**B**) Top 20 categories. (**C**) Landscape over time. The peak represents the number of occurrences of the discipline. The higher the peak, the more occurrences.

Data from the WoS show that the 2,664 articles cover 96 disciplines. We have built a network of disciplines involved in cardiac integrins through co-occurrence, cluster analysis, and timeline visualization, showing the evolution of mainstream and cross-cutting subjects. As shown in Figures 5A,B, the research area of cardiac integrins involves multiple disciplines, with “Cell Biology,” “Biochemistry, Molecular Biology,” and “Cardiac Cardiovascular Systems” being the three main disciplinary categories, reflecting the research content of integrins in the three dimensions of molecular, cellular and organ and acting as the basis and driving force for the mainstream and progression of research in the domain. The seven nodes marked with purple circles in the top 20 most frequent categories (Figure 5A) include “Cell Biology,” “Biochemistry Molecular Biology,” “Medicine Research Experimental,” “Physiology, Pharmacology Pharmacy,” “Immunology, Chemistry Multidisciplinary,” and “Cell Tissue Engineering,” which have a high BC (>0.1), indicating that these classifications are more relevant and communicative with other disciplines in the area of cardiac integrins.

The co-occurrence categories based on the log-likelihood ratio (LLR) can be divided into seven main categories, including #0 Cell Biology, #1 Materials Science, Multidisciplinary, #2 Cardiac & Cardiovascular Systems, #3 Reproductive Biology, #4 Engineering Biomedical, #5 Pharmacology Pharmacy, #6 Radiology, Nuclear Medicine & Medical Imaging, and the detailed information of clusters can be found in the (Supplementary material Table S1). In addition, according to the temporal evolution landscape (Figure 5C), we found that “Cell Biology” and “Materials Science” were two consistently popular categories during these 20 years, heralding continued advances in cardiac integrins in corresponding fields, such as the discovery of new functional and application technologies related to integrins (e.g., genetic engineering modification of extracellular vesicles) (12).

The “burst vocabulary” highlights the varying emphasis on different integrin themes throughout the course of the research (Table 2). Peripheral vascular disease (2003–2007), hematology (2005–2007), cardiac & cardiovascular systems (2005–2006), sport sciences (2005–2009), transplantation (2007–2013), multidisciplinary sciences (2012–2013), dentistry, oral surgery & medicine (2012–2017). radiology, nuclear medicine & medical imaging (2013–2016), nanoscience & nanotechnology (2015–2023), cell & tissue engineering (2015–2017), physics, applied (2016–2023), chemistry, physical (2017–2023), physics, condensed matter (2018–2023), chemistry, multidisciplinary (2018–2023), materials science, multidisciplinary (2019–2023), and developmental biology (2021–2023), all have high intensity (≥3), demonstrating popular disciplines involved in cardiac integrin at different phases of evolution or interactions with other disciplines.

**Table 2:** Top 16 subject categories with the strongest citation bursts in the field of cardiac IACs (2003–2023).

NO.	SC	Year	Strength	Begin	End	2003–2023
1	PERIPHERAL VASCULAR DISEASE	2003	8.51	**2003**	2007	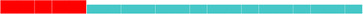
2	HEMATOLOGY	2003	9.2	**2005**	2007	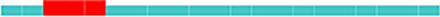
3	CARDIAC & CARDIOVASCULAR SYSTEMS	2003	3.83	**2005**	2006	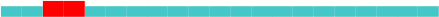
4	SPORT SCIENCES	2005	3.74	**2005**	2009	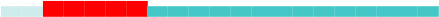
5	TRANSPLANTATION	2007	3.14	**2007**	2013	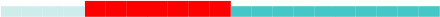
6	MULTIDISCIPLINARY SCIENCES	2003	6.36	**2012**	2013	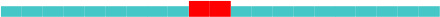
7	DENTISTRY, ORAL SURGERY & MEDICINE	2006	3.66	**2012**	2017	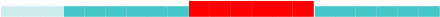
8	RADIOLOGY, NUCLEAR MEDICINE & MEDICAL IMAGING	2004	3.45	**2013**	2016	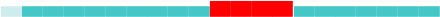
9	NANOSCIENCE & NANOTECHNOLOGY	2015	7.39	**2015**	2023	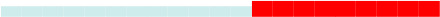
10	CELL & TISSUE ENGINEERING	2007	3.95	**2015**	2017	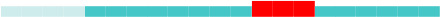
11	PHYSICS, APPLIED	2016	4.09	**2016**	2023	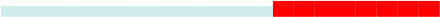
12	CHEMISTRY, PHYSICAL	2010	4.19	**2017**	2023	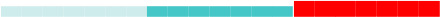
13	PHYSICS, CONDENSED MATTER	2016	3.5	**2018**	2023	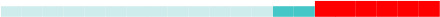
14	CHEMISTRY, MULTIDISCIPLINARY	2011	22.87	**2019**	2023	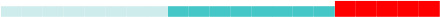
15	MATERIALS SCIENCE, MULTIDISCIPLINARY	2005	7.55	**2019**	2023	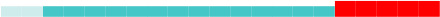
16	DEVELOPMENTAL BIOLOGY	2003	5.75	**2021**	2023	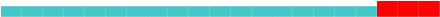

The bars represent years, with red bars representing years in which the burst terms lasted and blue bars representing years in which the burst terms did not last.

The bold values represent the years when the terms begin to “burst”.

### 3.5 Co-occurrence analysis of keywords

**Figure 6:**
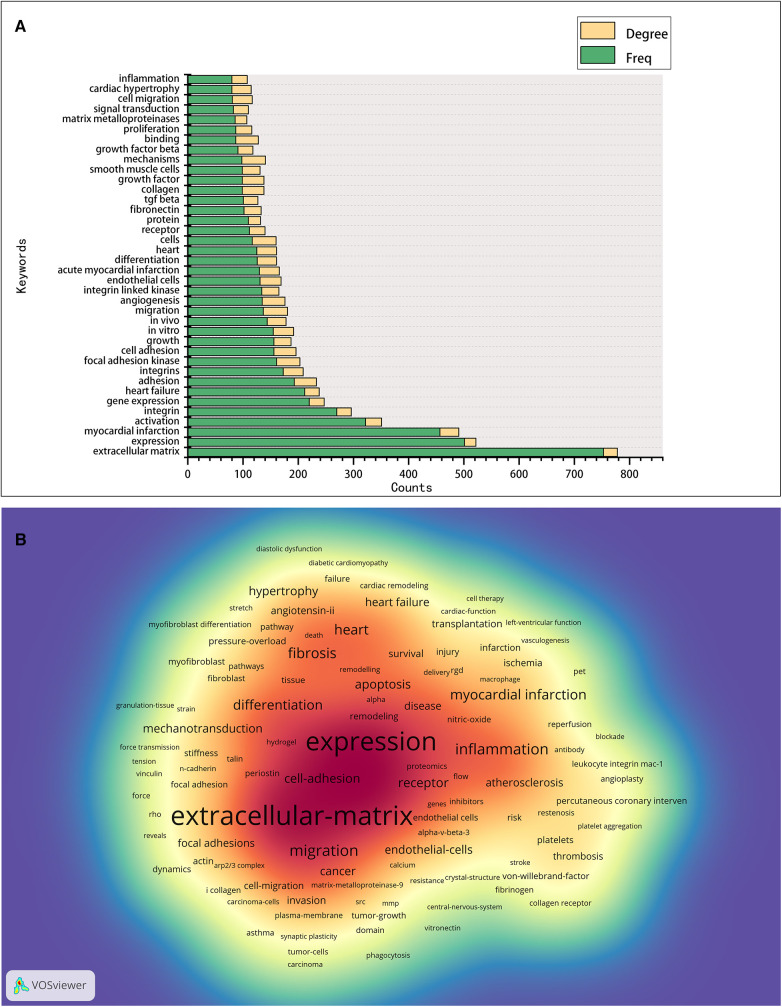
Co-occurrence analysis of keywords in the field of cardiac IACs (2003–2023). (**A**) Top 38 keywords. (**B**) Keyword density map: the redder the color, the higher the density and the more often the term occurs.

Keyword analysis aims to identify trends and hot topics, which is one of the key methods for tracking scientific developments. The WoS-based statistics show 655 keywords in 2,664 publications, of which 26 appear 100 times or more, and 57 appear 50 times or more (Figure 6). The 38 keywords with a frequency of 80 times or more are presented in Figure 6A, and the keyword density map is shown in Figure 6B. We found that the keyword with the largest nodes and the highest frequency was the “extracellular matrix,” with 753 occurrences, consistent with integrins' cell biological role. Concerning other keywords, some are the activities and mediated functions of cardiac integrins, like “migration,” “binding,” “adhesion,” “expression,” “signal transduction,” “angiogenesis,” “differentiation,” “proliferation,” and “growth,” the first three having a high degree. Some terms involve integrin-related proteins, interacting molecules, complex components, or downstream signals, such as “focal adhesion kinase,” “integrin-linked kinase,” “TGF beta,” “fibronectin,” and “matrix metalloproteinases.” Others are the predominant diseases, pathological changes, or expressions of different cell types associated with cardiac integrins, such as “myocardial infarction,” “heart failure,” “cardiac hypertrophy,” “endothelial cells,” and “smooth muscle cells.” Notably, the presence of the majority of keywords in 2003 suggests a level of maturity in the development of the field.

**Figure 7:**
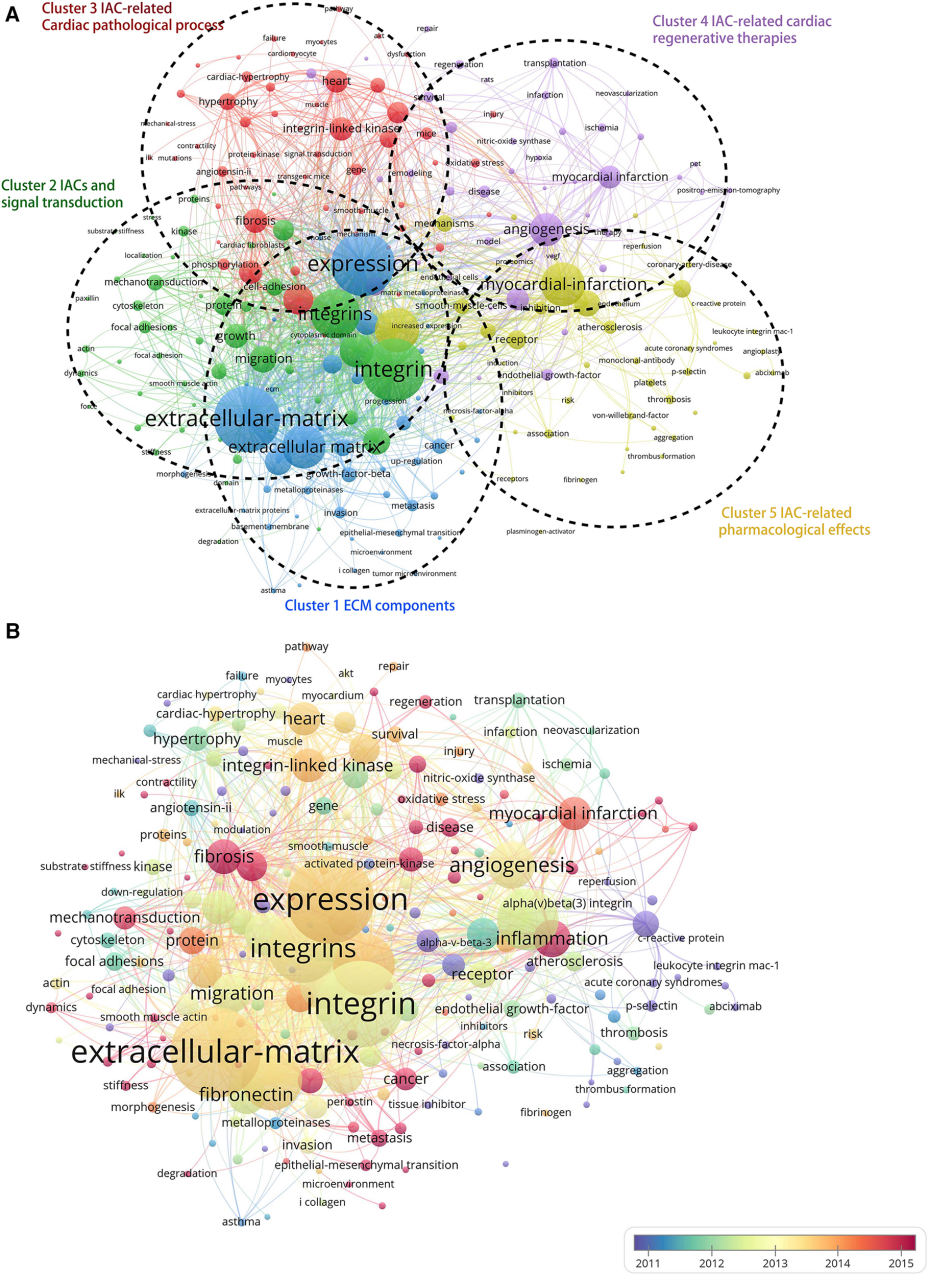
Cluster analysis and time-overlay visualization of keywords in the field of cardiac IACs (2003–2023). (**A**) Cluster analysis of keywords: the frequency is represented by point size; the keywords of research fields are divided into five clusters with different colors. (**B**) Time-overlay visualization map of the co-occurrence keywords; blue points represent earlier occurrences, and red points indicate later occurrences.

As shown in Figure 7A, the Vosviewer-based keyword co-occurrence demonstrates five main clusters: #1 IAC-related ECM components (e.g., extracellular matrix, metalloproteinase, growth factor beta, fibronectin, periostin), #2 IACs and signal transduction (e.g., foal adhesion, mechanotransduction, kinase, substrate stiffness, cytoskeleton, paxillin), #3 IAC-related cardiac pathological process (e.g., Cardiac hypertrophy, angiotensin II, remodeling, fibrosis, fibroblasts, heart failure), #4 IAC-related cardiac regenerative therapy (e.g., myocardial infarction, revascularization, PET, transplantation), and #5 IAC-related pharmacological effects (e.g., inhibitor, thrombosis, platelet, abciximab, monoclonal-antibody). In addition, keywords were color-coded differently using Vosviewer based on their average frequency of occurrence in all published articles (Figure 7B). The blue color indicates that the keyword appeared earlier, and the red color indicates that the keyword appeared later. Terms like “fibrosis,” “mechanotransduction,” “inflammation,” and “regeneration” have a higher density after 2015, indicating that relevant research areas would receive more focused attention in the future.

The term “burst vocabulary” refers to keywords that frequently appear over a while and can reflect the hot topics and their evolution of the relevant fields in the corresponding duration. Table 3 shows the top 25 burst keywords in the field of cardiac integrins from 2003 to 2023. More terms on different aspects of integrins were revealed, in addition to the keywords mentioned above: 2 silent polymorphisms (2003–2006), angioplasty (2003–2007), tissue inhibitor (2003–2005), endothelial progenitor cells (2007–2010), transplantation (2008–2015), β1 integrin (2013–2015), alpha(v)beta(3) integrin expression (2013–2017). mesenchymal stem cells (2014–2016), therapy (2016–2023), tenascin c (2019–2023), and inflammation (2020–2023) all have high intensity (≥5), indicating that they are hot topics and top rising topics in different development stages of cardiac integrins.

**Table 3:** Top 25 burst keywords in the field of cardiac IACs (2003–2023).

NO.	Keywords	Year	Strength	Begin	End	2003–2023
1	adhesion molecules	2003	7.31	**2003**	2008	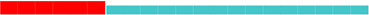
2	2 silent polymorphisms	2003	6.46	**2003**	2006	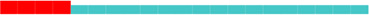
3	angioplasty	2003	5.96	**2003**	2007	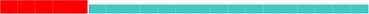
4	tissue inhibitor	2003	5.8	**2003**	2005	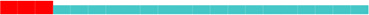
5	collagen receptor	2003	5.61	**2003**	2006	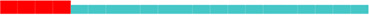
6	signal transduction	2003	10.5	**2004**	2009	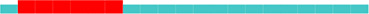
7	protein kinase	2004	6.76	**2004**	2006	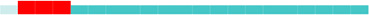
8	nitric oxide synthase	2005	7.01	**2005**	2010	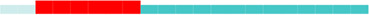
9	smooth muscle cells	2003	6.79	**2005**	2009	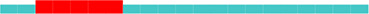
10	endothelial progenitor cells	2005	5.8	**2007**	2010	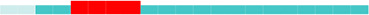
11	transplantation	2008	12.19	**2008**	2015	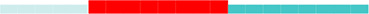
12	hypertrophy	2003	5.21	**2010**	2013	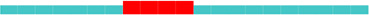
13	beta 1 integrin	2013	5.28	**2013**	2015	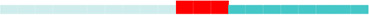
14	alpha(v)beta(3) integrin expression	2009	5.2	**2013**	2017	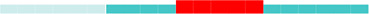
15	mesenchymal stem cells	2007	6.13	**2014**	2016	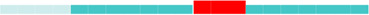
16	bone marrow	2008	5.38	**2014**	2018	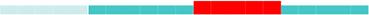
17	mechanotransduction	2013	6.51	**2015**	2017	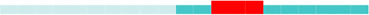
18	therapy	2014	6.6	**2016**	2023	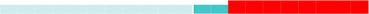
19	integrin activation	2009	5.68	**2016**	2021	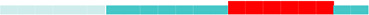
20	progression	2018	9.62	**2018**	2023	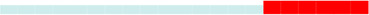
21	mechanisms	2003	7.67	**2018**	2023	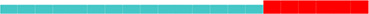
22	dysfunction	2010	6.25	**2018**	2023	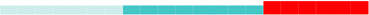
23	tumor microenvironment	2019	7.51	**2019**	2023	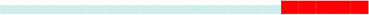
24	tenascin c	2005	7.1	**2019**	2023	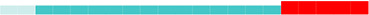
25	inflammation	2009	7.95	**2020**	2023	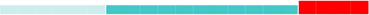

The bars represent years, with red bars representing years in which the burst terms lasted and blue bars representing years in which the burst terms did not last.

The bold values represent the years when the terms begin to “burst”.

### 3.6 Co-occurrence analysis of references

**Figure 8:**
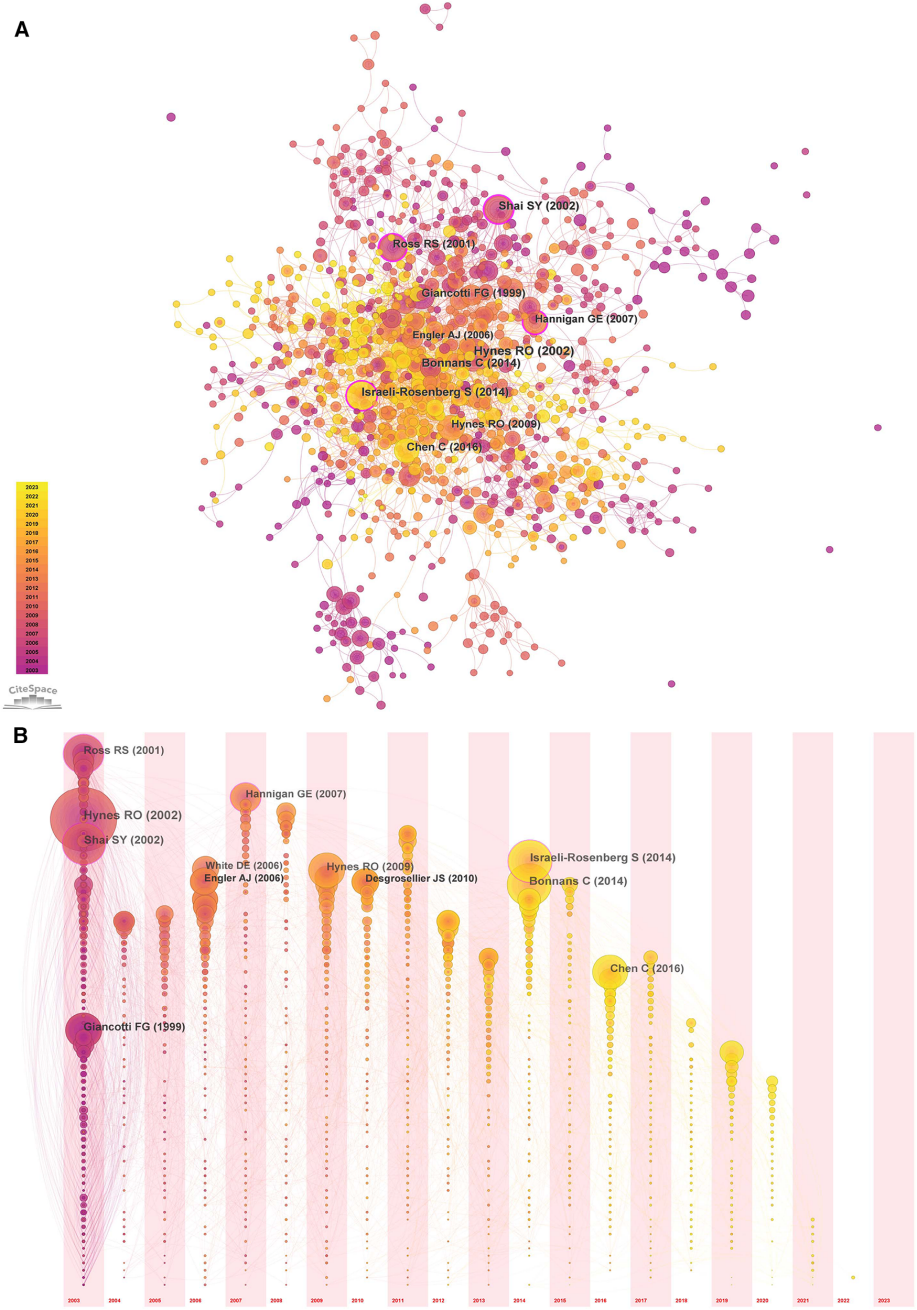
Co-occurrence analysis of references in the field of cardiac IACs (2003–2023). (**A**) Co-citation network; (**B**) Timezone view. The circular node size represents the number of citations in the literature. The larger the node, the more citations; the node color represents the year of publication. The darker the color, the older the year of publication; the purple circle outside the node, BC > 0.1.

**Figure 9:**
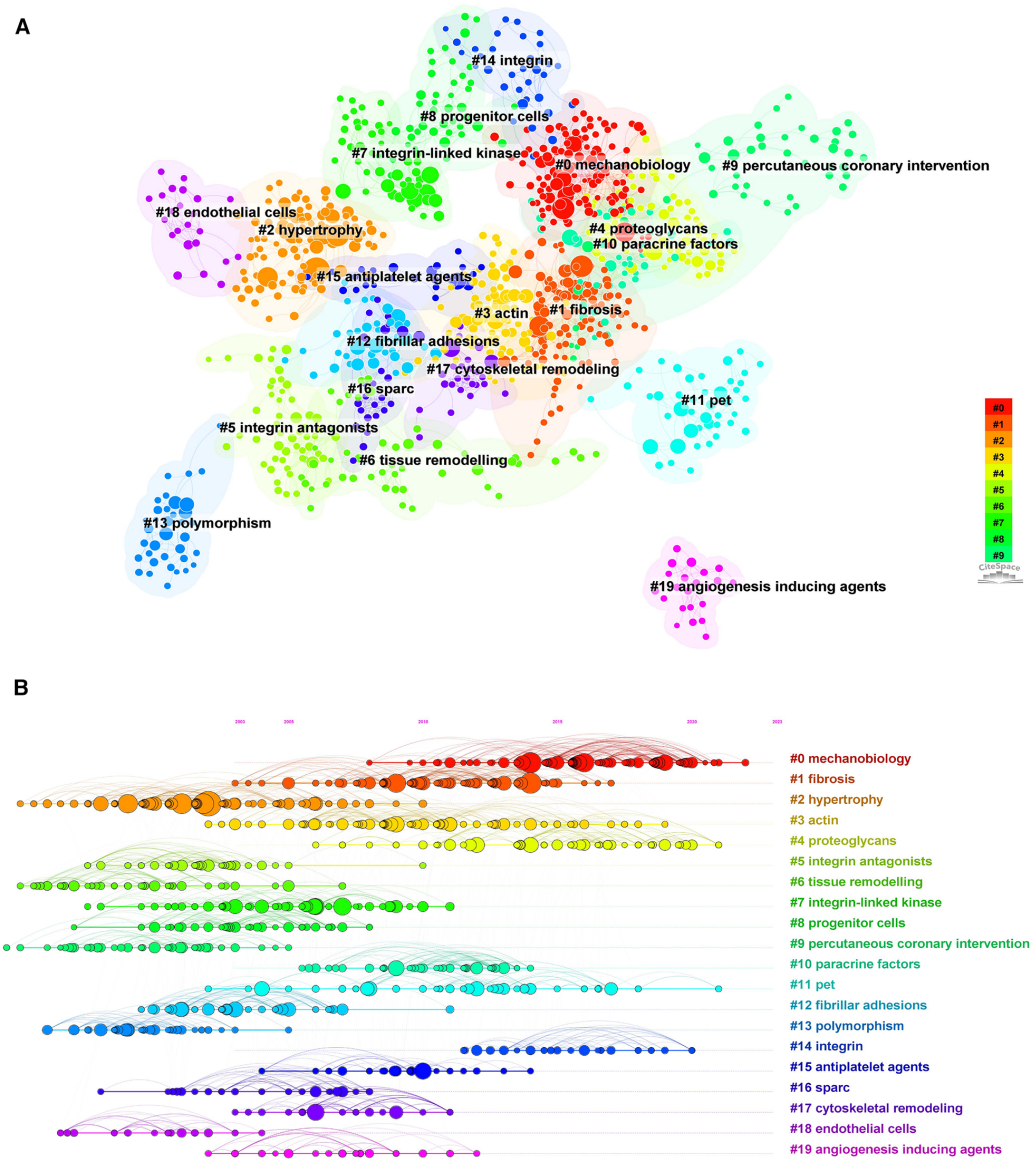
Cluster analysis and timeline view of references in the field of cardiac IACs (2003–2023). (**A**) Co-citation clustering; (**B**) Timeline view. Different colored nodes and zones represent different clusters, and the node size represents the number of citations in the literature. The larger the node, the more citations.

The cited literature serves as the basis and evidence for the research frontiers of a particular discipline. By conducting a thorough analysis of highly cited papers, one can gain a deeper understanding of the direction of the field and identify crucial evidence. Statistics from WoS show that the 1,452 cited papers involve 44,497 citations. Table 4 shows the top 10 highest co-cited literature. “Integrin signaling and the role of the extracellular matrix in development” were cited most frequently. In addition, papers on the discussion of myocardial integrin-related proteins and integrin-linked kinase (ILK) in cardiac contraction and repair, as well as the study of integrin β1 in myocardial development, had higher centrality (>0.1), suggesting a more important role and association of these three articles. Figures 8, 9 show the co-citation network, clustering, and timeline view. There are mainly 20 clusters, including #0 mechanobiology, #1 fibrosis,#2 hypertrophy, #3 actin, #4 proteoglycans, #5 integrin antagonists, #6 tissue remodeling, #7 integrin-linked kinase, #8 progenitor cells, #9 percutaneous coronary intervention, #10 paracrine factors,#11 PET, #12 fibrillar adhesions, #13 polymorphism, #14 integrin α, #15 antiplatelet agents, #16 sparc, #17 cytoskeletal remodeling, #18 endothelial cells, and #19 angiogenesis inducing agents.

**Table 4:** Top 10 co-cited references in the field of cardiac IACs (2003–2023).

NO	Count	Burst	Burst Begin	Burst End	Co-cited references titles	DOI
1	75	24.64	2004	2010	Integrins: bidirectional, allosteric signaling machines	10.1016/S0092-8674(02)00971-6
2	50	18.47	2016	2023	Remodeling the extracellular matrix in development and disease	10.1038/nrm3904
3	49	16.17	2015	2023	**Integrins and integrin-associated proteins in the cardiac myocyte**	10.1161/CIRCRESAHA.114.301275
4	48	14.08	2005	2009	**Cardiac myocyte-specific excision of the beta1 integrin gene results in myocardial fibrosis and cardiac failure.**	10.1161/hh0402.105790
5	45	14.75	2003	2009	Integrins and the myocardium	10.1161/hh1101.091862
6	41	14.8	2011	2017	The extracellular matrix: not just pretty fibrils	10.1126/science.1176009
7	41	17.85	2003	2007	Integrin signaling	10.1126/science.285.5430.1028
8	40	17.9	2018	2023	Integrins and integrin-related proteins in cardiac fibrosis	10.1016/j.yjmcc.2015.11.010
9	34	10.01	2008	2015	**Integrin-linked kinase at the heart of cardiac contractility, repair, and disease**	10.1161/01.RES.0000265233.40455.62
10	75	24.64	2011	2014	Integrins: bidirectional, allosteric signaling machines	10.1016/S0092-8674(02)00971-6

Bolded are the three publications (BC > 0.1) which have the purple circle outside the node in Figure 8.

We browsed the top 100 highly co-cited publications (see Supplementary material Table S2). Among these papers, the general functions of integrins and related proteins have been extensively studied and discussed, and details can be found in the excellent, highly cited reviews dedicated to the subject. The research at the molecular biology level covers bidirectional signal transduction, mechanistic signaling (13, 14), integrin activation (15), specific components (16), signaling (13, 17) and functional mapping (18) of IACs (3), and interactions with ECM factors (19) (e.g., fibronectin, matrix metalloproteinases, growth factors, cytokines, periostin, tenascin-C, and SPARC). Among these, focal adhesion kinase (FAK) and ILK are important components of IACs and play a central role in cardiac development, contraction, repair, and pathology (20–22). Various integrin-mediated functions at the cellular and tissue levels have also been discussed in depth, such as cell adhesion (23, 24), cell migration (25), matrix assembly (26), tissue remodeling (27), angiogenesis, and lymphangiogenesis (28). These are the basis for integrin-mediated cardiac embryonic development and repair after injury (29).

## 4 Discussion

### 4.1 Global trends

Integrin is a subject with a long history of research. From January 2003 to March 2023, the overall publication trend for cardiac integrins rose year on year, with 2,664 papers published in English, mainly involving two types of literature, articles, and reviews, accompanied by an increasing number of citations and attention. At the same time, given the long history of integrins as a research topic and the flattening growth rate of annual publication counts, as well as the presence of the majority of keywords already in 2003, the field of cardiac integrin research may have a high level of complexity and a certain maturity, suggesting its possible increased research intensity and difficulty. Based on present data, we have also predicted that more interest in this field will be gained in the coming years. The current optimistic results will also allow researchers to conduct further high-quality studies. In the statistical and co-occurrence analysis of countries, institutions, funding, journals, journals, and authors, we found that the United States, as well as the University of California System and the United States Department of Health Human Services from the United States, published the most papers, suggesting that the United States has the highest emphasis and contribution to research in this area. In addition, Elsevier, *Plos One*, and the author ROSS RS published the most papers, while *Cardiovascular Research* had the highest average number of citations per document (ACD), suggesting a concentration of high-quality research.

### 4.2 Discipline evolution

Based on the analysis of subject categories with co-occurrence, clustering, and temporal landscape evolution in the field of cardiac integrins, “Cell Biology,” “Biochemistry Molecular Biology,” and “Cardiac Cardiovascular Systems” are the three main underlying themes involved in cardiac integrins. Additionally, “Nanoscience & Nanotechnology,” “Chemistry, Physical,” “Physics, Condensed Matter,” “Materials Science,” “Multidisciplinary,” and “Developmental Biology” are popular disciplines that continue to date. This classification and clustering information represents important aspects involved in cardiac integrin research, such as the combined application of multidisciplinary techniques with physics, chemistry, materials, and computing, the intersection with regenerative medicine, immunology, imaging, hematology, oncology, and related drug development and technological innovation. Among them, “Developmental Biology” displays new and dramatic changes from 2021 to 2023, foreshadowing the focus and possible breakthrough of integrins in cardiac regenerative medicine, considering that regeneration/repair shares many pathways with organogenesis during embryonic development.

### 4.3 Research directions and hotspots

We identified the main research areas on integrins and IACs in cardiac repair/regeneration by constructing keyword co-occurrence networks and reading the top 100 highly cited literature on the subject. The research areas were categorized in the following directions: (1) extracellular matrix biochemical and biophysical signaling, (2) cardiac IAC composition, assembly, and function in cardiac regeneration, repair, and development, and (3) IAC-related cardiac regenerative therapies and pharmacological effects as targets.

#### 4.3.1 ECM components mediate cardiac development and repair via IACs

The components of the ECM, structural proteins (e.g., collagen) and non-structural proteins (e.g., growth factors, cytokines, matricellular proteins such as SPARC), as well as their physical properties, are discussed in the context of cardiac development and regeneration. Numerous studies have shown that the interaction of ECM elements and properties with integrins and focal adhesion (FA) proteins, as well as their downstream signaling, is essential for the development of the embryonic heart, such as mediating myocardial proliferation, survival, differentiation, and maturation, and the molecular biology involved can be used as a guide for cardiac regeneration and repair treatments. For instance, as components of the ECM secreted by embryonic cardiac fibroblasts, fibronectin (FN), collagen, and EGF-like growth factors act as specific signals to upregulate mitogen-activated protein kinase kinase (MEK)/extracellular regulated kinase 1/2 (ERK1/2) and phosphoinositide 3-kinase (PI3K)/protein kinase B (Akt) through the interaction between ECM/β1 integrin and EGF to mediate cardiomyocyte proliferation (30). Notably, β1 integrins and EGF receptors can form complexes at the cell membrane, induce EGF receptor tyrosine phosphorylation, and generate downstream signaling events, including cell survival and proliferation, in response to the ECM (31). Secreted protein, acidic and rich in cysteine (SPARC), is a matricellular protein that functions in the extracellular processing of newly synthesized collagen and can act by interacting with integrin dimers or by activating ILK. Fibroblasts isolated from SPARC null left ventricle (LV) showed significant differences in the expression of genes encoding ECM and adhesion molecule genes, including FN, connective tissue growth factor (CTGF; CCN2), matrix metalloproteinase-3 (MMP-3), and tissue inhibitor of metalloproteinase-2 (TIMP-2), accompanied by impaired fibroblast activation and adverse cardiac remodeling following MI (32). In contrast, adenoviral overexpression of SPARC in mice improved collagen maturation and prevented cardiac dilatation and dysfunction after MI (33). This suggests an essential role for SPARC in fibroblast activation, collagen deposition, and extracellular matrix remodeling after myocardial infarction.

#### 4.3.2 IACs mediate biomechanical signaling in cardiac repair/regeneration

As key downstream enzymes of the ECM-integrin transduction pathway, FAK and ILK also play critical roles in mediating cardiac development. For example, FAK inactivation in the embryonic heart manifests as a thin ventricular wall and ventricular septal defects (VSD), leading to a lethal embryonic phenotype. The mechanism is related to the reduced cardiomyocyte proliferation caused by FAK deficiency and defective regulation of MEF2a by the FAK-Src complex in cardiomyocytes (34). The latter is essential in the transcriptional regulation of myogenesis and morphogenesis (35). In addition, the Rho superfamily of small GTPases plays an essential role in integrin mechanistic signaling, as demonstrated by the regulation of cell adhesion, motility, and morphology through the regulation of cytoskeletal dynamics, including stress fibril assembly, actin polymerization, and FA formation. Members of the Rho superfamily of small GTPases and their downstream signaling have also been found to play important functions in cardiac embryonic development and cardiac repair. Recent studies show that substrate stiffness triggers translocation of vestigial-like family member 3 (VGLL3) in myofibroblasts to the nucleus via the integrin β1/Rho/ROCK/actin pathway, promoting myofibroblast collagen production in mouse and human fibrotic hearts (36). In post-MI studies, a collagen-derived peptide mimic of matrix cryptin (p1159) was found to increase cardiac fibroblast migration through activation of the RhoA pathway by the membrane receptor integrin α4, reduce adverse left ventricular remodeling, dilation, collagen deposition, fibrosis and increase local vascular networks after myocardial infarction by regulating fibrotic scar deposition, alignment and perfusion, and effectively improves cardiac systolic function (37). In addition, ROCK is a downstream effector of the Rho subfamily of small GTPases. Conditional activation of the ROCK2 kinase structural domain (ROCK2:ER) was found to increase non-myosin IIB (NM IIB) contraction in the postnatal heart, thereby altering the balance of cell-to-matrix adhesion and enhancing CM-ECM interactions (i.e., a5/β1-FN), ultimately promotes nuclear translocation of the mechanosensitive transcriptional co-activator yes-associated protein (Yap) and cardiomyocyte proliferation (38).

#### 4.3.3 Emerging applications of IACs in cardiac regenerative therapy

Based on the content of the most cited publications, statistical and visual data analysis results, and our expertise, we elaborate on the crucial concepts and findings underpinning the discipline. Different isoforms of integrins and their associated proteins and appropriate mechanical signaling can promote cardiac repair after myocardial injury, especially myocardial infarction, involving gene therapy (ILK, α7β1D), modified cardiac progenitor/stem cells, endothelial progenitor cell transplantation, bone marrow mesenchymal stem cells (BMSCs), outer matrix transplantation therapy (β1, ILK), cell-free therapy with stem cell-derived paracrine factors (thymosin β4, periostin), non-invasive assessment of angiogenic αvβ3 molecular imaging, and cardiac tissue engineering.

##### 4.3.3.1 Gene therapy

Significant clinical and conceptual advances in cardiovascular gene therapy have been made in the last 20 years and are used in multiple aspects of cardiovascular disease research (39). Preclinical model studies have found that ILK gene therapy and integrin α7β1D microinjection contribute to cardiac repair after ischaemic heart injury. As early as 2007, Gregory Hannigan et al. published a review proposing the conjecture that ILK might have a central role in mediating cardiac repair (21). Subsequently, several studies have highlighted that treatment with adenoviral vectors expressing ILK effectively preserved cardiac function and left ventricular geometry, accompanied by enhanced angiogenesis, increased cardiomyocyte proliferation, and reduced apoptosis and fibrosis in MI rats and swines induced by left anterior descending coronary artery (LAD), thus advancing the development of cardiac regenerative therapies (40, 41). In addition, integrin α7 binds to β1 to form a major laminin-binding receptor, which is highly expressed in adult cardiomyocytes. Hideshi Okada et al. (42) showed that cardiomyocyte-specific overexpression of integrin α7β1D induced by microinjection significantly reduced infarct size and protected against ischemia-reperfusion myocardial injury in mice. The mechanisms involved that α7β1D overexpression reduced the entry of excess Ca^2+^ into mitochondria, prevented the opening of the mitochondrial permeability transition pore (mPTP) after hypoxia/reoxygenation (H/R), and possibly mitigated the mitochondria-mediated necrotic pathway, and the effect of stabilizing the ryanodine receptor 2 (RyR2) interdomain interaction induced by β1D integrin.

##### 4.3.3.2 Paracrine factors

Besides the stem/progenitor cells themselves, the paracrine factors or exosomes secreted by these cells have been shown to drive multiple beneficial biological effects (11). The highly cited literature also reported the role of thymosin β4 on ILK and the resulting mediated protective effects against ischaemic heart disease. Thymosin β4, a major member of the β-thymosin family characterized by g-actin binding capacity (43), is one of the most secreted factors in mesenchymal stem cells and embryonic endothelial progenitor cells (eEPCs) (44). Thymosin β4 has been identified as a necessity for cardiac development and a candidate for the development of multipotent cardioprotective agents in adult cardiac ischemic events, including acute myocardial infarction, chronic ischemia, and ischemia-reperfusion injury (45). As early as 2004, Ildiko Bock-Marquette et al. found that subjects treated immediately after LAD with thymosin β4 thoracic injection showed significantly improved cardiac function, reduced cardiac dilatation, myocyte death and scar volume compared to controls; mechanistic studies showed that thymosin β4 formed a functional complex with the adaptor proteins PINCH and ILK in the cytoplasm, upregulating ILK and promoted phosphorylation of its downstream signal AKT, which positively affects cardiomyocyte and endothelial migration and survival. In particular, ILK, PINCH, and Parvin form a heterotrimeric complex called the IPP complex, which interacts with other components of the IACs to link integrin and actin cytoskeletons, coordinates signaling and gene transcription and regulates cytoskeletal dynamics and integrin activation, thus controlling multiple cellular responses, such as cell survival, proliferation, dynamics, and tissue (46).

As a non-structural ECM protein (47), periostin can be involved in the processes of tissue repair, remodeling, and fibrosis as an autocrine or paracrine factor via its FAS1 structural domain binding to integrins (e.g., αvβ3 and αvβ5) on the surface of target cells (48, 49). It is generally accepted that periostin is inextricably linked to cardiac myofibroblast function and the course of normal or pathological fibrosis and that it mediates valve maturation and the phenotypic conversion of mesenchymal progenitor cells to cardiac fibroblasts during cardiac development (49, 50). Studies suggested that periostin could stimulate FAK and AKT phosphorylation via αv integrin, promote migration and differentiation of cardiac fibroblasts towards the infarcted region, and thereby avert cardiac rupture by enhancing the stiffness of the left ventricular wall through collagen synthesis (51). Recent studies demonstrated that exosomes secreted from cardiac explant-derived progenitor cells (CPC) promote cardiomyocyte cell cycle-reentry via a short periostin isoform expressed on their surfaces, whereas recombinant full-length periostin does not; the mechanism of action is to promote FAK phosphorylation, actin polymerization and YAP nuclear translocation in cardiac myocytes (52). These findings reveal a potential role for short-length periostin in the repair of ischaemic myocardial injury.

##### 4.3.3.3 Cell transplantation

Cell transplantation therapy with ILK-modified cardiac progenitor cells (CPCs) and mesenchymal stem cells (MSCs) has been demonstrated to promote post-infarction angiogenesis, reduce fibrosis and apoptosis, thereby preserving left ventricular function and myocardial perfusion and shrinking infarct size (41, 53). CPCs overexpressed with ILK exhibited enhanced viability, proliferation, migration, and DNA synthesis, as well as upregulated expression of phosphorylated AKT (p-AKT) and cyclin D1. Cell therapies based on endothelial progenitor cells (EPCs) and their specific subpopulations (e.g., CACs) and cell-free therapies, like paracrine factors or extracellular vesicles, have also received extensive attention in cardiac repair, while integrin β and ILK signaling have been reported to be involved. Further exploration by Yujia Yue et al. found that knockdown of ILK in exosomes of inflamed EPCs inhibited NF-KB activation, attenuated the inflammatory response, and enhanced the repair activity of EPCs in the ischemic heart (54), suggesting that ILK is a potential key target for EPCs to mediate the repair of ischaemic myocardial injury. Moreover, *in vitro* and *in vivo* data showed that transplantation of integrin β1 overexpression-modified BMSCs significantly increased the adhesion of BMSCs and protected cardiomyocytes in a rat model of MI, inhibited apoptosis and regulated cell survival signaling by activating FAK and ILK, as evidenced by a decrease in the expression of the pro-apoptotic proteins cystein-3 and bax, and a significant increase in the expression of anti-apoptotic proteins such as bcl-2 in the myocardium, thereby contributing to angiogenesis and cardiac survival (55). Treatment with bone marrow-derived circulating angiogenic cells (CACs) + collagen matrix demonstrated better efficacy in improving myocardial perfusion, ejection fraction, ventricular wall thickness preservation, glucose uptake, and vessel density compared to cell or matrix transplantation alone, largely through integrin α2/ILK signaling-mediated CAC-matrix interaction and integrin α5/ERK-dependent upregulation of integrin α5 (56, 57). This suggests that injectable delivery matrices are expected to enhance engraftment and the overall efficacy of cardiac cell therapies. In addition, the role of integrin-related signaling in improving metabolic pathways is of interest.

##### 4.3.3.4 Non-invasive assessment of angiogenesis

In the LAD-induced MI rat model, integrin β3 localizes to the vasculature in the peri-infarct region in a temporally coordinated manner, as evidenced by a significant increase in its protein levels on day 3, remaining elevated over the course of the 4-week observation (58). This indicates that cardiac integrin β3 may promote angiogenesis in the peri-infarct region as part of the remodeling process. Angiogenesis and arteriogenesis are essential for the repair of damaged myocardium and the prognosis of MI and are also considered to be one of the hallmarks and phenomena that precede myocardiogenesis (59). αvβ3 integrins are expressed at low levels by endothelial cells in the resting state and are significantly upregulated on activated endothelial cells in the angiogenic state (60). However, αvβ3 is thought to have both possible positive and negative modulatory effects on angiogenesis in different biological contexts (61). Thus, αvβ3 expression is currently more predominantly explored for non-invasive assessment of angiogenesis post-MI and molecular imaging of oncological (62) instead of being an interventional target for drugs that modulate angiogenesis. The development of this technology has facilitated the evaluation of therapeutic strategies to stimulate cardiac angiogenesis after MI, such as cytokine therapy, gene therapy, and cell transplantation (63, 64). It can also help predict left ventricular repair, remodeling, and prognosis in MI and permit risk stratification of patients following MI. Common imaging techniques include positron emission tomography (PET) and single-photon emission computed tomography (SPECT) imaging with radionuclides, magnetic resonance imaging (MRI)/computed tomography (CT) imaging, ultrasound imaging, etc (65–68).

##### 4.3.3.5 Tissue engineering

During normal cardiac development, changes in the composition and properties of the myocardial ECM are coordinated with the expression of specific integrins. In addition to biochemical signals of ECM origin, microenvironmental biomechanical signals, including dimensionality, rigidity, and spatial arrangement of the ECM (e.g., matrix structure), have an impact on cardiomyocyte proliferation, differentiation, and maturation, which is discussed intensively in several highly cited reviews (29, 69). Mechanical matrix features regulate cellular tension, and force transmission pathways are provided by integrin-associated adhesion. For example, stiff substrates increase the expression of FA components, including non-muscle α-actin, filament, talin, and FAK (70), causing downstream pathways like PI3K/AKT, Wnt signaling, and the p38 Mitogen-activated protein kinase (MAPK)/c-Jun N-terminal kinase (JNK) pathway to be upregulated in response to dynamic stiffness, thereby mediating myocardial development and differentiation (71). Increased force significantly prolongs the lifetime of integrin bonds, induces FA and stress fiber formation, allows force to reach the nucleus via actomyosin contractility, and promotes the release of transcriptional regulators such as YAP/TAZ from the nucleus, ultimately altering cell cycle and proliferation (13). Multiple *in vitro* biomaterial approaches that mimic the cardiomyocyte microenvironment have been developed and designed to explore cardiac development and repair in cardiac tissue engineering. For instance, AuNP–Col scaffolds with nanoscale stiffness changes promote cardiac intercalated discs assembly through the β1 integrin-mediated ILK/AKT/GATA4 signaling pathway (72); PEG hydrogels with high concentrations of laminin and RGD-binding integrins promote the production of cardiomyocyte-like cells through a combination of improved fibroblast reprogramming efficiency and increased proliferation (73).

### 4.4 Research frontiers and future landscapes

Burst keywords represent emerging trends and research frontiers. As shown in Table 3, we used the burst detection function of CiteSpace to identify burst keywords in these publications and found two current research hotspots in Tenasin-C (2019–2023) and inflammation (2020–2023). We highlight the emerging interest in the role of both matricellular proteins, represented by Tenasin-C, and integrin-related proteins in the regulation of inflammation. Tenasin-C, a large extracellular matrix (ECM) glycoprotein hexameric multidomain protein that interacts with integrin dimers, has been found to accelerate adverse ventricular remodeling, heart failure, and fibrosis in residual myocardium after MI through a variety of pathways (74). For example, tenasin-C enhanced the inflammatory response via integrin αVβ3/FAK-Src/NF-*κ*B by accelerating macrophage migration and pro-inflammatory/pro-fibrotic cytokine synthesis, leading to increased cardiac fibrosis. In contrast, collagen fibril deposition, macrophage accumulation, and cytokine expression in the perivascular region were significantly reduced in tenasin-C knockout/angiotensin II mice (75). This suggests that targeting tenasin-C is a possible pharmacological target to promote cardiac repair after myocardial injury by ameliorating inflammation.

Notably, macrophage infiltration and migration into the injured myocardium after myocardial infarction also involves the upregulation of integrins and adhesion molecules, particularly β1 and β3, which are highly expressed isoforms in macrophages (76). Silencing of integrin β1 inhibited macrophage migration. In contrast, downregulation and internalization of integrin β1 at the macrophage plasma membrane activates downstream FAK/Src signaling (77). At the same time, the FAK-Src complex mediates phosphorylation of two scaffolding molecules, pilein and p130 crk-associated substrate (CAS), regulating the actin cytoskeleton and thus promoting macrophage migration (78). Early resident macrophage recruitment after myocardial infarction has been found to accelerate angiogenesis and tissue repair and improve cardiac remodeling and function. Thus, pharmacological targeting of macrophage integrins for internalization and recirculation and manipulation of their migration and function may be a promising emerging therapeutic strategy to optimize the infarct repair process.

### 4.5 Strengths and limitations

In order to obtain reliable and objective results, this study was conducted on the WoSCC database. The bibliometric and visual analysis was performed by various software such as Prism 8, Origin 2021, Vosviewer, and Citespace to reliably and objectively evaluate the current status and trends of IACs in cardiac repair/regeneration research. However, our study still has some limitations. It is well known that publications from different databases (e.g., WoS, Pubmed, Embase, and Cochrane Library) vary. Therefore, we may have missed some publications due to database bias. In addition, due to the limitations of the English search strategy of the SCI-extended databases, non-English literature may be missed, leading to language bias. Finally, there is no standardized parameter setting for Vosviewer and Citespace, and there may be differences between the statistical results of the two software and WoS, so the analysis results may differ between software.

## 5 Conclusion

To date, the role of integrin-related proteins and IACs in cardiac repair/regeneration has not been systematically summarised. This study shows the global status and trends of IACs in cardiac repair/regeneration. The United States is the largest contributor to the study and is leading this area of research globally. The journal *Circulation Research* attracts the largest number of high-quality publications related to this field. We predict more interest in IACs in cardiac repair/regeneration in the coming years. More notably, the rapidly emerging role of matricellular proteins (e.g., tenasin-C) and non-protein components of the ECM (e.g., extracellular vesicles, non-coding RNAs) in regulating matrix structure and function may be a further breakthrough point in the future; the emerging role of IACs and their downstream molecular signaling in cardiac repair are also of significant interests, such as induction of cardiac proliferation, differentiation, maturation, and metabolism, fibroblast activation and inflammatory modulation through the induction of macrophage migration and polarization.

## Data Availability

The original contributions presented in the study are included in the article/**Supplementary Material**, further inquiries can be directed to the corresponding author.
