# Supplementary material for: Global research trends and emerging opportunities for integrin adhesion complexes in cardiac repair: a scientometric analysis
Source: Front Cardiovasc Med. 2024 Apr 18;11:1308763. doi: 10.3389/fcvm.2024.1308763 (PMC11063371; doi:10.3389/fcvm.2024.1308763)
Supplement: Supplementary file 1 [file Datasheet1.docx]

**1. Search strategy**

1#=((((((((((((((((TS=(cardiac remodeling)) OR TS=(Ventricular Remodeling)) OR TS=(Myocardial Remodeling)) OR TS=(extracellular matrix remodeling)) OR TS=(cardiac hypertrophy)) OR TS=(Ventricular hypertrophy)) OR TS=(Ventricular dilatation)) OR TS=(Myocardial hypertrophy)) OR TS=(Cardiomyocyte hypertrophy)) OR TS=(Cardiac fibrosis)) OR TS=(Myocardial fibrosis)) OR TS=(Cardiomyocyte death)) OR TS=(Cardiomyocyte apoptosis)) OR TS=(Cardiomyocyte necrosis)) OR TS=(myocardial infarction )) OR TS=(Cardiac repair)) OR TS=(Heart failure)

2#=(TS=(Integrin*))

3#=1# AND 2#

**2. Table. S1. The detailed information of clusters of Subject Categories**

| **ClusterID** | **Size** | **Silhouette** | **Mean**  **(Year)** | **Label (LLR)** | **Label (MI)** |
| --- | --- | --- | --- | --- | --- |
| 0 | 21 | 0.763 | 2005 | CELL BIOLOGY (288.17, 1.0E-4); BIOCHEMISTRY & MOLECULAR BIOLOGY (252.82, 1.0E-4); PERIPHERAL VASCULAR DISEASE (187.31, 1.0E-4); CARDIAC & CARDIOVASCULAR SYSTEMS (182.09, 1.0E-4); MEDICINE, RESEARCH & EXPERIMENTAL (92.36, 1.0E-4) | PSYCHOLOGY, DEVELOPMENTAL (2.77); DENTISTRY, ORAL |
| 1 | 17 | 0.853 | 2012 | MATERIALS SCIENCE, MULTIDISCIPLINARY (162.7, 1.0E-4); NANOSCIENCE & NANOTECHNOLOGY (115.37, 1.0E-4); CHEMISTRY, MULTIDISCIPLINARY (107.17, 1.0E-4); CHEMISTRY, PHYSICAL (101.69, 1.0E-4); PHYSICS, APPLIED (69.09, 1.0E-4) | PHYSICS, ATOMIC, MOLECULAR & CHEMICAL (0.3); ENGIN |
| 2 | 14 | 0.669 | 2004 | CARDIAC & CARDIOVASCULAR SYSTEMS (387.87, 1.0E-4); PERIPHERAL VASCULAR DISEASE (326.17, 1.0E-4); PHYSIOLOGY (198.01, 1.0E-4); CELL BIOLOGY (193.31, 1.0E-4); BIOCHEMISTRY & MOLECULAR BIOLOGY (126.53, 1.0E-4) | ALLERGY (1.21); NURSING (1.21); PEDIATRICS (1.21); |
| 3 | 12 | 0.788 | 2008 | REPRODUCTIVE BIOLOGY (138.39, 1.0E-4); DEVELOPMENTAL BIOLOGY (87.65, 1.0E-4); OBSTETRICS & GYNECOLOGY (61.23, 1.0E-4); PARASITOLOGY (33.75, 1.0E-4); VETERINARY SCIENCES (26.95, 1.0E-4) | INFECTIOUS DISEASES (0.12); TOXICOLOGY (0.12); ELE |
| 4 | 10 | 0.813 | 2008 | ENGINEERING, BIOMEDICAL (125.01, 1.0E-4); CELL & TISSUE ENGINEERING (119.11, 1.0E-4); BIOTECHNOLOGY & APPLIED MICROBIOLOGY (58.83, 1.0E-4); BIOPHYSICS (52.25, 1.0E-4); HEMATOLOGY (45.96, 1.0E-4) | ELECTROCHEMISTRY (0.82); CHEMISTRY, ANALYTICAL (0. |
| 5 | 8 | 0.754 | 2004 | PHARMACOLOGY & PHARMACY (196.05, 1.0E-4); CHEMISTRY, MEDICINAL (75.13, 1.0E-4); INTEGRATIVE & COMPLEMENTARY MEDICINE (40.58, 1.0E-4); OPHTHALMOLOGY (40.58, 1.0E-4); CELL BIOLOGY (26.66, 1.0E-4) | ELECTROCHEMISTRY (0.12); CHEMISTRY, ANALYTICAL (0. |
| 6 | 5 | 0.913 | 2007 | RADIOLOGY, NUCLEAR MEDICINE & MEDICAL IMAGING (106.08, 1.0E-4); BIOCHEMICAL RESEARCH METHODS (77.35, 1.0E-4); MATHEMATICAL & COMPUTATIONAL BIOLOGY (16.69, 1.0E-4); CELL BIOLOGY (11.83, 0.001); ACOUSTICS (8.32, 0.005) | CELL BIOLOGY (0.12); BIOCHEMISTRY & MOLECULAR BIOL |

**3. Fig. S1. The overlay visualization of journals with global publications about IACs in Cardiac repair (2003-2023). Node size represents the number of publications, the larger the node, the more publications; node color represents the year of publication,** **the redder the node, the newer the publication.**

**
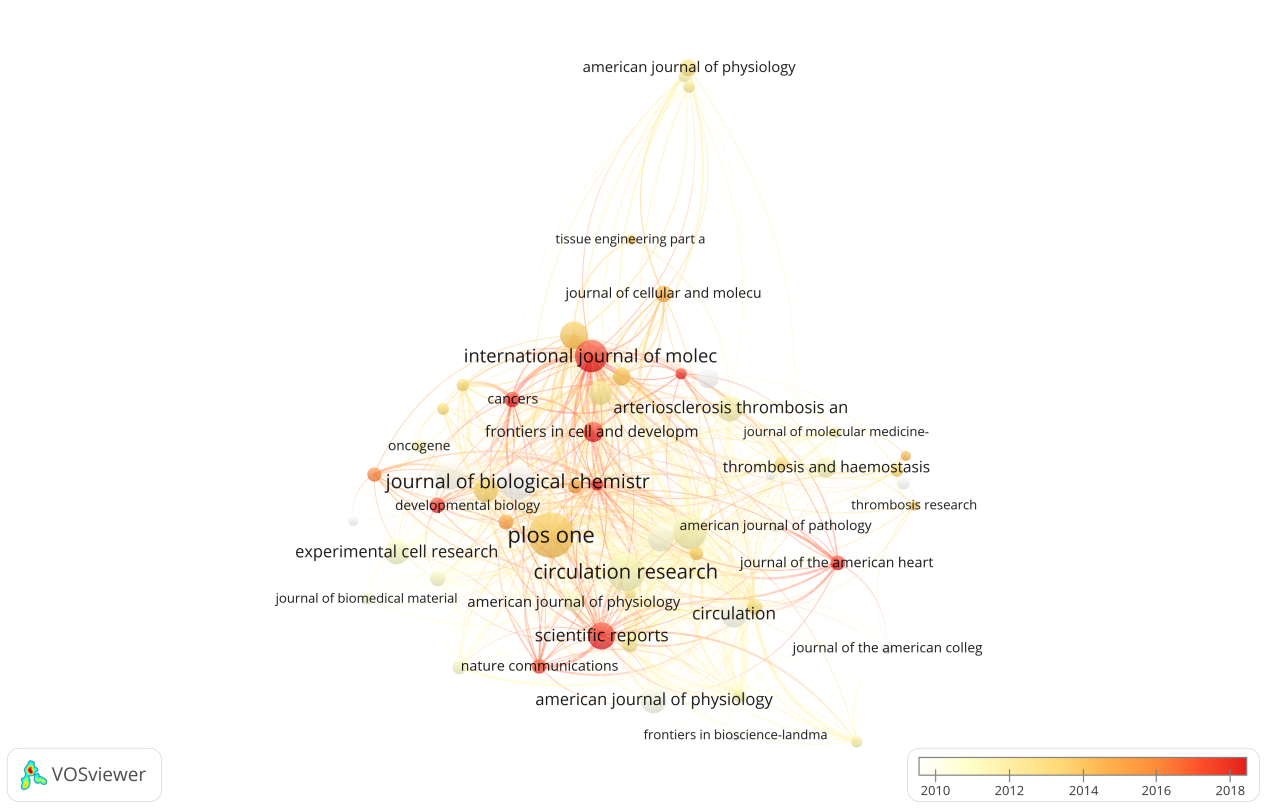
**

**4.** **Fig. S2.** **The IF and document number of journals with global publications about IACs in Cardiac repair (2003-2023): Size, Feq (the larger the size, the greater the number); Color, year (the darker the node, the later the publication).**


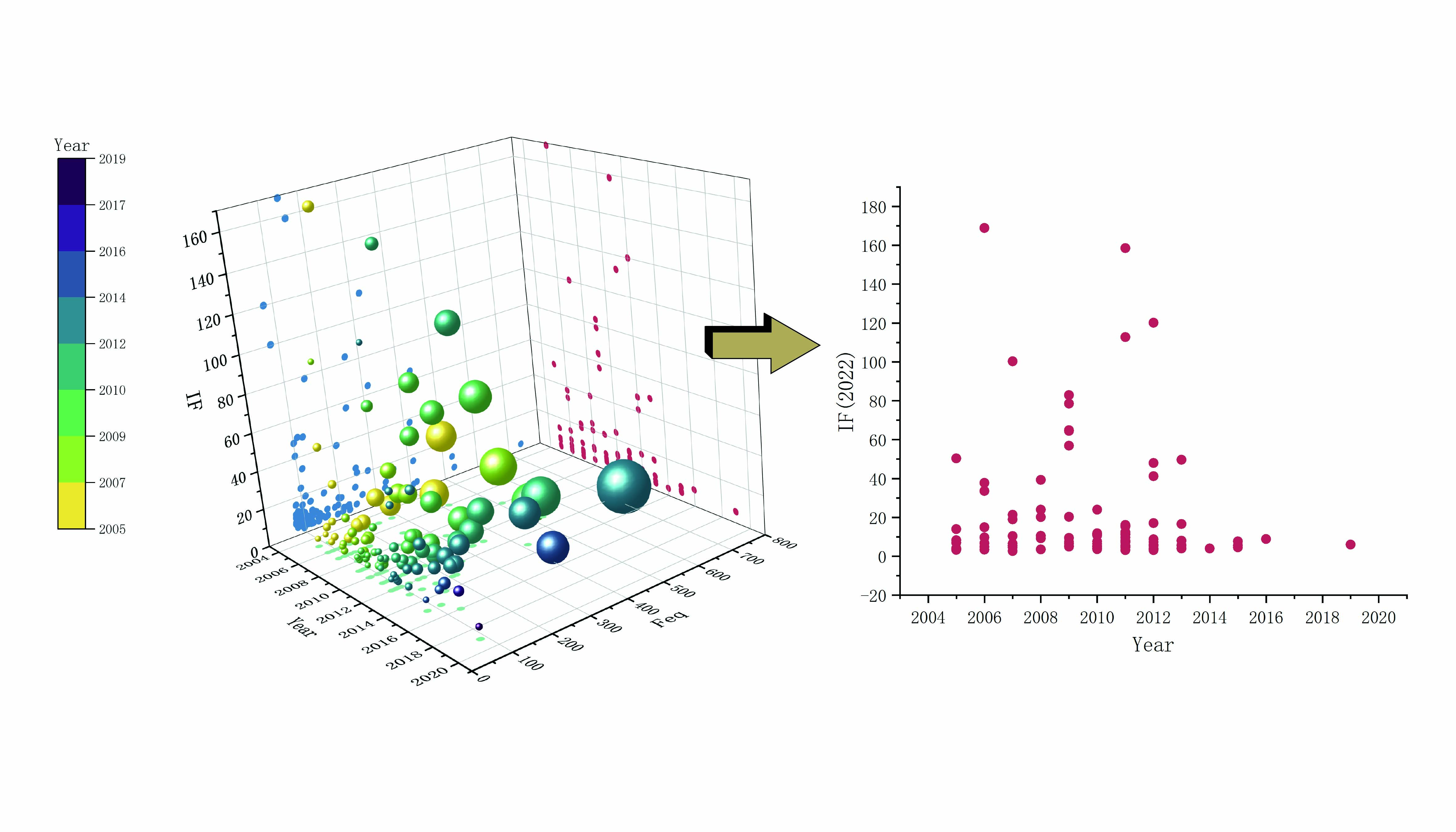


**5.Table. S2. Top 100 cited publications.**

| NO. | Freq | Burst | Title | DOI |
| --- | --- | --- | --- | --- |
| 1 | 75 | 24.64 | Integrins: bidirectional, allosteric signaling machines | 10.1016/S0092-8674(02)00971-6 |
| 2 | 50 | 18.47 | Remodelling the extracellular matrix in development and disease | 10.1038/nrm3904 |
| 3 | 49 | 16.17 | Integrins and integrin-associated proteins in the cardiac myocyte | 10.1161/CIRCRESAHA.114.301275 |
| 4 | 48 | 14.08 | Cardiac myocyte-specific excision of the beta1 integrin gene results in myocardial fibrosis and cardiac failure | 10.1161/hh0402.105790 |
| 5 | 45 | 14.75 | Integrins and the myocardium | 10.1161/hh1101.091862 |
| 6 | 41 | 14.8 | The extracellular matrix: not just pretty fibrils | 10.1126/science.1176009 |
| 7 | 41 | 17.85 | Integrin signaling | 10.1126/science.285.5430.1028 |
| 8 | 40 | 17.9 | Integrins and integrin-related proteins in cardiac fibrosis | 10.1016/j.yjmcc.2015.11.010 |
| 9 | 34 | 10.01 | Integrin-linked kinase at the heart of cardiac contractility, repair, and disease | 10.1161/01.RES.0000265233.40455.62 |
| 10 | 32 | 11.42 | Matrix elasticity directs stem cell lineage specification | 10.1016/j.cell.2006.06.044 |
| 11 | 31 | 11.16 | Integrins in cancer: biological implications and therapeutic  opportunities | 10.1038/nrc2748 |
| 12 | 30 | 11.64 | Targeted ablation of ILK from the murine heart results in dilated cardiomyopathy and spontaneous heart failure | 10.1101/gad.1458906 |
| 13 | 29 | 9.67 | Targeted ablation of ILK from the murine heart results in dilated cardiomyopathy and spontaneous heart failure | 10.1101/gad.1448306 |
| 14 | 27 | 14.27 | Integrins as biomechanical sensors of the microenvironment | 10.1038/s41580-019-0134-2 |
| 15 | 27 | 10.73 | Matrix crosslinking forces tumor progression by enhancing integrin signaling | 10.1016/j.cell.2009.10.027 |
| 16 | 27 | 10.47 | Integrin-linked kinase expression is elevated in human cardiac hypertrophy and induces hypertrophy in transgenic mice | 10.1161/CIRCULATIONAHA.106.642330 |
| 17 | 26 | 10.09 | The extracellular matrix: a dynamic niche in cancer progression | 10.1083/jcb.201102147 |
| 18 | 26 | 8.78 | Integrins αvβ5 and αvβ3 promote latent TGF-β1 activation by human cardiac fibroblast contraction | 10.1093/cvr/cvu053 |
| 19 | 25 | 11.39 | Integrin-mediated mechanotransduction | 10.1083/jcb.201609037 |
| 20 | 25 | 8.24 | Cell migration: integrating signals from front to back | 10.1126/science.1092053 |
| 21 | 25 | 9.93 | Cardiac fibroblasts regulate myocardial proliferation through beta1 integrin signaling | 10.1016/j.devcel.2008.12.007 |
| 22 | 24 | 6.59 | Melusin, a muscle-specific integrin beta1-interacting protein, is required to prevent cardiac failure in response to chronic pressure overload | 10.1038/nm805 |
| 23 | 24 | 11.5 | Beta1 integrins participate in the hypertrophic response of rat ventricular myocytes | 10.1161/01.RES.82.11.1160 |
| 24 | 24 | 8.15 | Noninvasive imaging of myocardial angiogenesis following experimental myocardial infarction | 10.1172/JCI200420352 |
| 25 | 23 | 9.18 | Transmembrane crosstalk between the extracellular matrix--cytoskeleton crosstalk | 10.1038/35099066 |
| 26 | 23 | 7.89 | Molecular imaging of early αvβ3 integrin expression predicts long-term left-ventricle remodeling after myocardial infarction in rats | 10.2967/jnumed.111.091652 |
| 27 | 22 | 9.95 | Taking cell-matrix adhesions to the third dimension | 10.1126/science.1064829 |
| 28 | 22 | 8.35 | Mechanotransduction and extracellular matrix homeostasis | 10.1038/nrm3896 |
| 29 | 22 | 9.51 | Targeting of αv integrin identifies a core molecular pathway that regulates fibrosis in several organs | 10.1038/nm.3282 |
| 30 | 22 | 7.71 | In vivo molecular imaging of angiogenesis, targeting alphavbeta3 integrin expression, in a patient after acute myocardial infarction | 10.1093/eurheartj/ehn129 |
| 31 | 21 | 7.19 | Integrin signalling: the tug-of-war in heart hypertrophy | 10.1016/j.cardiores.2005.12.015 |
| 32 | 21 | 9.1 | Striated muscle-specific beta(1D)-integrin and FAK are involved in cardiac myocyte hypertrophic response pathway | 10.1152/ajpheart.2000.279.6.H2916 |
| 33 | 20 | 7.75 | Tensional homeostasis and the malignant phenotype | 10.1016/j.ccr.2005.08.010 |
| 34 | 20 | 7.74 | Integrins | 10.1007/s00441-009-0834-6 |
| 35 | 20 | 7.63 | Hallmarks of cancer: the next generation | 10.1016/j.cell.2011.02.013 |
| 36 | 20 | 6.75 | Analysis of the myosin-II-responsive focal adhesion proteome reveals a role for β-Pix in negative regulation of focal adhesion maturation | 10.1038/ncb2216 |
| 37 | 19 | 6.82 | Mechanically activated integrin switch controls alpha5beta1 function | 10.1126/science.1168441 |
| 38 | 19 | 9.09 | Association of the Platelet Glycoprotein Ia C807T Gene Polymorphism With Nonfatal Myocardial Infarction in Younger Patients | 10.1182/blood.V93.8.2449.408k34_2449_2453 |
| 39 | 19 | 5.46 | Inactivation of focal adhesion kinase in cardiomyocytes promotes eccentric cardiac hypertrophy and fibrosis in mice | 10.1172/JCI24497 |
| 40 | 19 | 8.69 | Thymosin beta4 activates integrin-linked kinase and promotes cardiac cell migration, survival and cardiac repair | 10.1093/cvr/cvn033 |
| 41 | 18 | 6.3 | Environmental sensing through focal adhesions | 10.1038/nrm2593 |
| 42 | 18 | 7.06 | Thymosin beta4 activates integrin-linked kinase and promotes cardiac cell migration, survival and cardiac repair | 10.1038/nature03000 |
| 43 | 18 | 8.78 | Definition of a consensus integrin adhesome and its dynamics during adhesion complex assembly and disassembly | 10.1038/ncb3257 |
| 44 | 18 | 8.61 | Association of two silent polymorphisms of platelet glycoprotein Ia/IIa receptor with risk of myocardial infarction: a case-control study | 10.1016/S0140-6736(98)06448-4 |
| 45 | 17 | 5.57 | The extracellular matrix modulates the hallmarks of cancer | 10.15252/embr.201439246 |
| 46 | 17 | 6.44 | Integrin activation and focal complex formation in cardiac hypertrophy | 10.1074/jbc.M006124200 |
| 47 | 17 | 6.1 | Increased expression of integrin-linked kinase attenuates left ventricular remodeling and improves cardiac function after myocardial infarction | 10.1161/CIRCULATIONAHA.109.870725 |
| 48 | 16 | 7.65 | Nucleotide Polymorphisms in the a 2 Gene Define Multiple Alleles That Are Associated With Differences in Platelet a 2b 1 Density | 10.1182/blood.V92.7.2382 |
| 49 | 16 | 7.58 | Cardiac αVβ3 integrin expression following acute myocardial infarction in humans | 10.1136/heartjnl-2016-310115 |
| 50 | 16 | 6.71 | Nanoscale architecture of integrin-based cell adhesions | 10.1038/nature09621 |
| 51 | 15 | 7.31 | Signal transduction via integrin adhesion complexes | 10.1016/j.ceb.2018.08.004 |
| 52 | 15 | 5.81 | Matricellular proteins in cardiac adaptation and disease | 10.1152/physrev.00008.2011 |
| 53 | 15 | 7.31 | Integrin activation by talin, kindlin and mechanical forces | 10.1038/s41556-018-0234-9 |
| 54 | 15 | 5.08 | Matrix metalloproteinase inhibitors and cancer: trials and tribulations | 10.1126/science.1067100 |
| 55 | 15 | 6.64 | Myofibroblasts and mechano-regulation of connective tissue remodelling | 10.1038/nrm809 |
| 56 | 15 | 6.26 | Mechanical regulation of a molecular clutch defines force transmission and transduction in response to matrix rigidity | 10.1038/ncb3336 |
| 57 | 15 | 5.5 | Temporal response and localization of integrins beta1 and beta3 in the heart after myocardial infarction: regulation by cytokines | 10.1161/01.CIR.0000051363.86009.3C |
| 58 | 14 | 5.58 | Focal adhesion kinase: the first ten years | 10.1242/jcs.00373 |
| 59 | 14 | 6.8 | Integrins in angiogenesis and lymphangiogenesis | 10.1038/nrc2353 |
| 60 | 14 | 7.5 | The extracellular matrix at a glance | 10.1242/jcs.023820 |
| 61 | 14 | 6.12 | Endothelial extracellular matrix: biosynthesis, remodeling, and functions during vascular morphogenesis and neovessel stabilization | 10.1161/01.RES.0000191547.64391.e3 |
| 62 | 14 | 5.3 | Mechanical stress-induced cardiac hypertrophy: mechanisms and signal transduction pathways | 10.1016/S0008-6363(00)00076-6 |
| 63 | 14 | 6.06 | Disruption of integrin function in the murine myocardium leads to perinatal lethality, fibrosis, and abnormal cardiac performance | 10.1016/S0002-9440(10)64055-2 |
| 64 | 14 | 6.04 | β1- and αv-class integrins cooperate to regulate myosin II during rigidity sensing of fibronectin-based microenvironments | 10.1038/ncb2747 |
| 65 | 14 | 7.61 | Moderated estimation of fold change and dispersion for RNA-seq data with DESeq2 | 10.1186/s13059-014-0550-8 |
| 66 | 14 | 7.01 | β1 integrin gene excision in the adult murine cardiac myocyte causes defective mechanical and signaling responses | 10.1016/j.ajpath.2011.12.007 |
| 67 | 13 | 6.36 | How matrix metalloproteinases regulate cell behavior | 10.1146/annurev.cellbio.17.1.463 |
| 68 | 13 | 0 | Concepts of extracellular matrix remodelling in tumour progression and metastasis | 10.1038/s41467-020-18794-x |
| 69 | 13 | 6.33 | The biology and function of fibroblasts in cancer | 10.1038/nrc.2016.73 |
| 70 | 13 | 5.03 | Cell adhesion: integrating cytoskeletal dynamics and cellular tension | 10.1038/nrm2957 |
| 71 | 13 | 6.42 | Regulation of cardiac hypertrophy by intracellular signalling pathways | 10.1038/nrm1983 |
| 72 | 13 | 4.52 | Periostin induces proliferation of differentiated cardiomyocytes and promotes cardiac repair | 10.1038/nm1619 |
| 73 | 13 | 5.91 | Genetic lineage tracing defines myofibroblast origin and function in the injured heart | 10.1038/ncomms12260 |
| 74 | 13 | 6.82 | Comparison of cyclic RGD peptides for αvβ3 integrin detection in a rat model of myocardial infarction | 10.1186/2191-219X-3-38 |
| 75 | 13 | 5.02 | Force sensing by mechanical extension of the Src family kinase substrate p130Cas | 10.1016/j.cell.2006.09.044 |
| 76 | 13 | 4.92 | Platelet glycoprotein IIb/IIIa integrin blockade with eptifibatide in coronary stent intervention: the ESPRIT trial: a randomized controlled trial | 10.1001/jama.285.19.2468 |
| 77 | 13 | 5.61 | GDF-15 is an inhibitor of leukocyte integrin activation required for survival after myocardial infarction in mice | 10.1038/nm.2354 |
| 78 | 12 | 6.44 | Cell Adhesion by Integrins | 10.1152/physrev.00036.2018 |
| 79 | 12 | 6.42 | Cell-matrix adhesion | 10.1002/jcp.21237 |
| 80 | 12 | 5.9 | Costameres, focal adhesions, and cardiomyocyte mechanotransduction | 10.1152/ajpheart.00749.2005 |
| 81 | 12 | 6.91 | Extracellular matrix and cell signalling: the dynamic cooperation of integrin, proteoglycan and growth factor receptor | 10.1530/JOE-10-0377 |
| 82 | 12 | 6.44 | Cardiac fibrosis: Cell biological mechanisms, molecular pathways and therapeutic opportunities | 10.1016/j.mam.2018.07.001 |
| 83 | 12 | 4.64 | Fibronectin fibrillogenesis, a cell-mediated matrix assembly process | 10.1016/j.matbio.2005.06.008 |
| 84 | 12 | 5.83 | Integrin ligands at a glance | 10.1242/jcs.03098 |
| 85 | 12 | 7.08 | Functional atlas of the integrin adhesome | 10.1038/ncb0807-858 |
| 86 | 12 | 4.97 | The matrix reorganized: extracellular matrix remodeling and integrin signaling | 10.1016/j.ceb.2006.08.009 |
| 87 | 12 | 6.5 | The ins and outs of fibronectin matrix assembly | 10.1242/jcs.00670 |
| 88 | 12 | 5.9 | Focal adhesion kinase: in command and control of cell motility | 10.1038/nrm1549 |
| 89 | 12 | 3.52 | Matrix metalloproteinases and the regulation of tissue remodelling | 10.1038/nrm2125 |
| 90 | 12 | 4.61 | Integrins protect cardiomyocytes from ischemia/reperfusion injury | 10.1172/JCI64216 |
| 91 | 12 | 5.77 | Talin binding to integrin beta tails: a final common step in integrin activation | 10.1126/science.1086652 |
| 92 | 12 | 5.2 | Application of (68)Ga-PRGD2 PET/CT for αvβ3-integrin imaging of myocardial infarction and stroke | 10.7150/thno.8809 |
| 93 | 12 | 6.07 | Double-blind, randomized trial of an anti-CD18 antibody in conjunction with recombinant tissue plasminogen activator for acute myocardial infarction: limitation of myocardial infarction following thrombolysis in acute myocardial infarction (LIMIT AMI) study | 10.1161/hc4801.100236 |
| 94 | 12 | 6.5 | Noninvasive imaging of angiogenesis with a 99mTc-labeled peptide targeted at alphavbeta3 integrin after murine hindlimb ischemia | 10.1161/CIRCULATIONAHA.104.485029 |
| 95 | 11 | 5.36 | Every step of the way: integrins in cancer progression and metastasis | 10.1038/s41568-018-0038-z |
| 96 | 11 | 5.29 | Adhesion-dependent cell mechanosensitivity | 10.1146/annurev.cellbio.19.111301.153011 |
| 97 | 11 | 5 | Tumour exosome integrins determine organotropic metastasis | 10.1038/nature15756 |
| 98 | 11 | 5.58 | Fibronectins in vascular morphogenesis | 10.1007/s10456-009-9136-6 |
| 99 | 11 | 5.9 | Extracellular matrix structure | 10.1016/j.addr.2015.11.001 |
| 100 | 11 | 4.4 | Quantitative proteomics of the integrin adhesome show a myosin II-dependent recruitment of LIM domain proteins | 10.1038/embor.2011.5 |
